# Combination of Sodium Butyrate and Immunotherapy in Glioma: regulation of immunologically hot and cold tumors via gut microbiota and metabolites

**DOI:** 10.3389/fimmu.2025.1532528

**Published:** 2025-04-14

**Authors:** Sui Li, Li Wang, MingYu Han, Huali Fan, Hailin Tang, Huile Gao, Guobo Li, Zheng Xu, Zhaokai Zhou, JunRong Du, Cheng Peng, Fu Peng

**Affiliations:** ^1^ West China School of Pharmacy, Sichuan University, Chengdu, China; ^2^ State Key Laboratory of Southwestern Chinese Medicine Resources, School of Pharmacy, Chengdu University of Traditional Chinese Medicine, Chengdu, China; ^3^ Bioinformatics Department, Jiangsu Sanshu Biotechnology Co., Ltd., Nantong, China; ^4^ State Key Laboratory of Oncology in South China, Guangdong Provincial Clinical Research Center for Cancer, Sun Yat-Sen University Cancer Center, Guangdong, China; ^5^ State Key Laboratory of Biotherapy, West China Hospital, Sichuan University, Chengdu, China; ^6^ Department of Urology, The First Affiliated Hospital of Zhengzhou University, Department of Clinical Medicine, Zhengzhou University, Henan, China; ^7^ Key Laboratory of Drug-Targeting and Drug Delivery System of the Education Ministry, Sichuan Engineering Laboratory for Plant-Sourced Drug and Sichuan Research Center for Drug Precision Industrial Technology, West China School of Pharmacy, Sichuan University, Chengdu, China

**Keywords:** anti-tumor immunity, gut microbiota and metabolites, glioma, sodium butyrate, PD-1/PD-L1, PI3K/Akt signaling pathway

## Abstract

**Background:**

Recent studies have highlighted the importance of cross-talk along the gut-brain axis in regulating inflammatory nociception, inflammatory responses, and immune homeostasis. The gut microbiota, particularly its bacterial composition, plays a crucial role in the development and function of the immune system. Moreover, metabolites produced by the gut microbiota can significantly impact both systemic immune responses and central nervous system (CNS) immunity. Sodium butyrate is a key metabolite produced by the gut microbiota and, as a histone deacetylase inhibitor, can enhance the anti-tumor immunity of cytotoxic CD8^+^ T cells. However, it remains unclear whether sodium butyrate treatment can enhance the efficacy of PD-1 blockade in glioma therapy. In this research, the effect and underlying mechanism of combination of gut microbiota metabolites and anti-mouse PD-1 mAb on glioma has been investigated.

**Methods:**

RNA-seq assay in glioma cell and biomedical databases, including ONCOMINE, GEPIA and TCGA were incorporated. Subsequently, the inhibitory effect of sodium butyrate on glioma cells and its related mechanisms were assessed through Counting Kit-8 (CCK-8), Flow Cytometry, Western blot (WB), reverse transcription-quantitative polymerase chain reaction (RT-qPCR), and other *in vitro* experiments. *In vitro*, an orthotopic mouse glioma model was established. MRI imaging, Immunohistochemistry, and Immune cell flow cytometry were used to investigate the therapeutic effects of combined sodium butyrate and PD-1 inhibitor treatment on glioma-bearing mice.

**Results:**

We discovered that deacetylation-associated gene expression is significantly increased in glioma patients and affects patient survival time. Moreover, we found sodium butyrate promoted glioma cell apoptosis, disrupted the cell cycle, and inhibited tumor growth. Additionally, sodium butyrate may upregulate PD-L1 expression in glioma cells by modulating the PI3K/AKT pathway. The experimental results demonstrated that this combination therapy significantly reduced tumor volume and prolonged survival in an orthotopic murine glioma model. Moreover, combination therapy led to an increase in the proportion of probiotic bacteria in the mouse gut microbiota, resulting in elevated levels of antitumor metabolites and a decrease in metabolites that affect immune cell function.

## 1 Introduction

Glioma is considered one of the most malignant tumors affecting the central nervous system and has high mortality and recurrence rates (1–3). Although PD-L1 is generally considered an immunosuppressive molecule, its expression may not always signify tumor immune evasion and could instead reflect an ongoing antitumor immune response (4). In glioma, which is classified as a “cold” tumor with fewer CD3 T cells, a greater frequency of myeloid cells (monocytes, macrophages, and microglia), and lower expression of PD-L1 and PD-1-expressing TILs (5–7), a phase III clinical trial of PD-1 for glioma revealed an overall treatment response rate of only 8% and did not significantly prolong the overall survival of glioma patients (8). Therefore, enhancing PD-L1 expression to increase T cell infiltration may offer a potential strategy for converting gliomas from ‘cold’ tumors to ‘hot’ tumors.

Recent studies have established a connection between the gut microbiome and the efficacy of immune checkpoint inhibitors (9–11). The potential mechanisms through which the gut microbiota mediates anti-tumor immunity involve the activation of T cell responses specific to microbial antigens. These responses either support tumor-specific immune activation or may cross-react with tumor-specific antigens (12, 13). However, it remains unclear how metabolites produced by the gut microbiota regulate anti-tumor immunity, and whether they can modulate tumor-infiltrating T cell responses in glioma.

In this study, we report that the sodium butyrate acted as gut microbiota metabolites to suppress glioma cell growth while increasing PD-L1 expression in glioma cells by activating the PI3K/AKT pathway. When combined with an anti-mouse PD-1 mAb, sodium butyrate resulted in tumor regression and an increased antitumor immune response in an orthotopic murine glioma model. Mechanistically, combination therapy enhanced immune cytokine levels and T-cell infiltration while also influencing the gut microbiota and its metabolites in mice.

## 2 Materials and methods

### 2.1 Cell culture

The human cell line U251 and the murine cell line GL261 were purchased from Zhong Qiao Xin Zhou Biotechnology (Shanghai, China). All cell lines were authenticated using STR profiling and cultured in DMEM (Gibco, MA, United States) supplemented with 10% fetal bovine serum (Gibco, MA, United States) and 1% penicillin/streptomycin (Invitrogen, MA, United States).

### 2.2 Sodium butyrate

Sodium butyrate was purchased from Sigma. Aliquots of DMSO-reconstituted Sodium butyrate were stored at -80°C. For *in vitro* studies, stocks were diluted to the final concentration immediately prior to use. For *in vivo* use, sodium butyrate was dissolved and sonicated in sterile PBS.

### 2.3 CCK-8 assay

Cells were seeded into 96-well plates (1×10^5^ cells/well) and cultured until cell attachment. After sodium butyrate administration for 48 h, 10 μL of Cell Counting Kit-8 (CCK-8) reagent (Dojindo, Japan) was added to each well, and the plates were incubated for 1 h. The absorbance of each well was measured at 450 nm using a 96-well plate reader (Thermo Fisher Scientific, USA).

### 2.4 Flow cytometry analyses

After the administration of sodium butyrate and 48 hours of incubation, apoptosis and the cell cycle distribution were analyzed using flow cytometry. For cell apoptosis analysis, the cells were collected and stained with Annexin V-FITC and propidium iodide (PI) in the dark following the procedure of the Apoptosis Detection Kit from Solarbio (China). For cell cycle analysis, the cells were collected and fixed in 70% ethanol for 2 hours at 4°C. Subsequently, the cells were stained with RNase and PI reagent in the dark following the protocol of the Cell Cycle Analysis Kit from Beyotime (China). After incubation, the cells were analyzed using a flow cytometer (FACSCelesta, BD, USA).

The tumor tissues were processed mechanically to obtain single-cell suspensions, and the cell concentration was adjusted to 10^6^ cells/mL using PBS. For each tissue, two flow cytometry tubes were prepared. To each tube, 100 µL of the single-cell suspension was added along with the fluorochrome-conjugated mAbs specific for mouse CD3, CD4, CD8, and Tregs (BD Pharmingen, USA). Tumor A: CD3, CD4, CD8, PD-L1, B220, Live-dead. Tumor B: CD4, CD25, Live-dead. The tubes were incubated at 4°C in the dark for 30 minutes. After incubation, tube A was washed with PBS, then resuspended in 400 µL of PBS and analyzed using the FACSCanto Flow Cytometer (BD Biosciences). Tube B was subjected to fixation and permeabilization following the procedure outlined in the reagents manual. Following fixation and permeabilization, 1 µg each of FoxP3 and IFN-γ antibodies (both from BD Pharmingen) were added, mixed, and incubated at 4°C in the dark for 30 minutes. After incubation, 400 µL of PBS was added to re-suspend the cells, and immediate analysis was conducted using the FACSCanto Flow Cytometer (BD Biosciences). The data were acquired using a FACSCanto Flow Cytometer (BD Biosciences) and analyzed using FlowJo software (TreeStar, V7.6.5 for Windows).

### 2.5 Mouse models and drug administration

7 week male C57BL/6 mice (20-22g) were purchased from Beijing Huhukang Laboratory Animal Company (Beijing, China). For the *in vivo* study, mice were orthotopically implanted with 1×10^5^ GL261 glioma cells. Assessment of tumor growth and survival was conducted after daily intraperitoneal administration of 1.2 g/kg sodium butyrate (Sigma, USA), either alone or in combination with 100 µg of PD-1 blocking antibody from BioXCell twice weekly, for a total of 4 weeks, starting on the seventh day after GL261 inoculation. The treatment control group received sterile PBS. The mice were euthanized 28 days after inoculation. All animal experiments adhered to the regulations of the ethics committee of the Experimental Animal Administration of Sichuan University.

### 2.6 Magnetic resonance imaging

Cerebral magnetic resonance imaging (MRI) was conducted on a small animal 7 T MRI scanner (Time Medical, Shanghai, China) *in vivo*. Every mouse was given anesthesia with 2% isoflurane administered through inhalation. Imaging was performed using a 2D T2-weighted sequence in the axial orientation. Tumor volumes were calculated using ITK-SNAP program.

### 2.7 Western blot

Total proteins were extracted with RIPA buffer (Beyotime, Shanghai, China) according to the manufacturer’s instructions. The lysates were then subjected to SDS–PAGE and transferred to a PVDF membrane (Bio-Rad, CA, United States). Primary antibody incubation was carried out overnight at 4°C. Antibodies against p-PI3K, PI3K, p-AKT, AKT, survivin, Cyclin A2, Cyclin B1, cdc2, p-cdc2, cdc25C, HDAC1, HDAC2, HDAC3 and HDAC6 were purchased from Cell Signaling Technology (Shanghai, China). Subsequently, the immunoblots were incubated with the appropriate secondary antibody, detected using ECL Plus Reagent (Beyotime, Shanghai, China) and quantified using ImageJ software.

### 2.8 Quantitative real-time PCR

Cells or tissues were lysed using TRIzol from Invitrogen (Thermo Fisher Scientific, MA, United States). RNA was isolated from the samples using a standard phenol–chloroform separation protocol, and cDNA was generated with an iScript kit from Bio-Rad. Expression levels were assessed by quantitative real-time PCR using the SYBR Green platform on a Bio-Rad CFX96 system. Real-time polymerase chain reaction (PCR) was conducted following previous methods, and the data were processed using the 2^−ΔΔCT^ method (14). 18S ribosomal RNA was used as the reference gene in all experiments. Primers were designed using NCBI-Blast listed in **Supplementary Table S1**.

### 2.9 Histological and immunohistochemical analyses

Paraffin-embedded tissues were cut into 3-μm-thick sections. Hematoxylin and eosin (HE) staining was carried out following standard histological protocols. For immunohistochemistry (IHC), the slides were incubated with the following primary antibodies from Abcam (Cambridge, United Kingdom) after blocking: Ki-67 (ab16667), CD4 (ab133616), and CD8 (ab217344). Quantification of the IHC results was performed by counting the number of immunostaining-positive cells using Image-Pro Plus software.

### 2.10 Gene expression profiling interactive and molecular docking analysis

The mRNA sequencing data of the HDAC family together with its corresponding clinical information of glioma patients were retrieved from GEPIA (http://gepia2.cancer-pku.cn/), a web-based interactive tool providing comprehensive and customizable analyses with The Cancer Genome Atlas (TCGA) and Genotype-Tissue Expression Project (GTEx) RNA sequencing data as resources.

To improve the prediction of the relationship between Sodium butyrate and PI3K/AKT and HDAC related genes in the protein-protein interaction (PPI) analysis, we performed molecular association prediction. First, we obtained the structure of the gene from the Protein Data Bank (PDB) (http://www.rcsb.org) and acquired the 3D structure of Sodium butyrate from the PubChem database (https://pubchem.ncbi.nlm.nih.gov/). Next, we removed the hydrogen and ligands and dehydrated the hub gene proteins using the PyMOL 1.7.x software (http://www.pymol.org/). The AutoDock Tools 1.5.6 software (https://autodock.scripps.edu/) was used to convert the file to the PDBQT format. The PyMOL software was used to visualize the docking results.

### 2.11 RNA sequencing and single-cell transcriptomic analysis

The cells were incubated with sodium butyrate for 48 h, after which total RNA was isolated using TRIzol reagent (Invitrogen). Subsequently, RNA sequencing (RNAseq) was performed using a commercially available service (service ID# F18FTSSCWLJ1284, BGI, Huada Gene, Wuhan, China). In brief, total RNA was fragmented into short fragments, and mRNA was enriched using oligo (dT) magnetic beads. This was followed by cDNA synthesis, and the double-stranded cDNA was purified and enriched through PCR amplification. The library products were then sequenced using BGIseq-500. For the data analysis, KEGG pathway and GO bioinformatics analyses were conducted by BGI utilizing the Dr. TOM approach, an in-house customized data mining system of BGI. The altered (upregulated or downregulated) expression of genes is presented as log2FC, representing the log-transformed fold change (log2FC = log2[B] - log2[A], where A and B represent the gene expression values under different treatment conditions). Single-cell sequencing data of orthotopic glioma in mice was downloaded from the GEO database (GSE246154) and analyzed using the R Programming Language.

### 2.12 Gut microbial 16S RNA sequencing and metabolite analysis

Fecal genomic DNA was extracted using a fecal DNA extraction kit (TIANGEN, Beijing, China) following the manufacturer’s instructions. We weighed 25 mg of the Fecal into a centrifuge tube and added 400 µL of a methanol/acetonitrile mixture along with magnetic beads for sample disruption. The mixture was centrifuged at 25,000 rpm for 2 minutes and set aside for later use. A mixed standard solution of seven SCFAs was prepared and serially diluted (CAL1-CAL12). A 20 µL aliquot of the sample and 60 µL of the standard solution were mixed with 60 µL of cold methanol/acetonitrile (2:1), vortexed for 5 minutes, and then incubated at -20°C for 4 hours. The mixture was centrifuged at 20,000 g at 4°C for 15 minutes, and 40 µL of the supernatant was transferred to an EP tube. To the EP tube containing 40 µL of the supernatant, we added 20 µL of 200 mM 3-NPH (solvent: 50% acetonitrile) and 20 µL of a 120 mM EDC-6% pyridine mixture (solvent: 50% acetonitrile). The reaction was incubated in a metal bath at 40°C with shaking for 30 minutes, then cooled to room temperature on ice and briefly centrifuged. After derivatization, 80 µL of 1000 Da internal standard (solvent: 10% acetonitrile) was added and mixed well. In a filtration plate, 90 µL of water and 90 µL of the derivatized sample were added. The mixture was centrifuged at 3,000 g at 4°C for 5 minutes. The filtered supernatant (90 µL) was transferred to a new 96-well plate, and 10 µL was injected for LC-MS/MS (Waters I-Class system coupled with an AB Sciex 6500 mass spectrometer). MRM transitions (ion pairs) were automatically identified and integrated using default parameters in MultiQuant software (SCIEX, USA), with manual verification to ensure accuracy. The concentration of short-chain fatty acids (ng/mg) was calculated using the formula:FACs Concentration (ng/mg)= (C*V)/M, where C is the concentration of the target analyte (ng/mL), V is the volume of the extracted sample (µL), and M is the weight of the sample (mg).

The near-full-length 16S rRNA genes were amplified using the 27F/1390R primers. Subsequently, the constructed library was sequenced on the PacBio Sequel platform. The high-quality reads obtained were analyzed using QIIME software. The qualified sequences were then clustered into operational taxonomic units (OTUs) at 97% similarity. Metabolic extracts from fecal samples were analyzed by gas chromatography–mass spectrometry (GC–MS) based on a previous research protocol (15). All the analyses were conducted using BMKCloud (www.biocloud.net).

### 2.13 Statistical analysis

The values presented in the study represent the mean ± SEM of at least three independent experiments. All the statistical analyses were conducted using SPSS 25.0 and GraphPad Prism 5.0 software. The log-rank test was used to analyze Kaplan–Meier survival curves. For comparisons between two groups, two-tailed Student’s t tests were used. Comparisons among multiple groups were performed using one-way analysis of variance (ANOVA), followed by the least significant difference (LSD) *post hoc* test. Differences with a *p* value less than 0.05 were considered to indicate statistical significance.

## 3 Results

### 3.1 Sodium butyrate inhibits glioma cell proliferation and promotes U251 cell apoptosis

**Figure 1:**
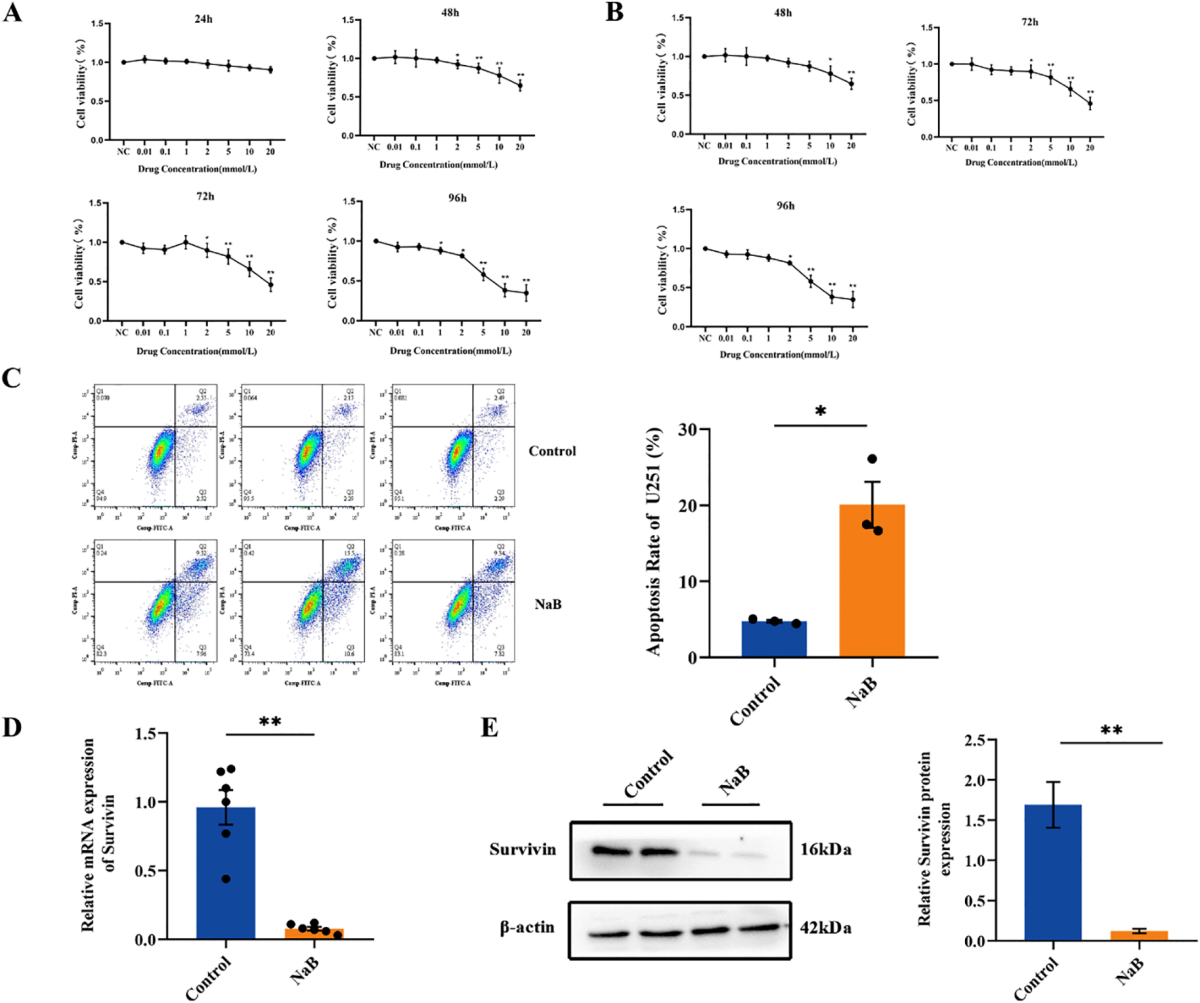
Sodium butyrate inhibits glioma cell proliferation and promotes U251 cell apoptosis. **(A)** U251 and **(B)** GL261 glioma cell viability was examined using the CCK-8 assay. The data are expressed as the mean ± SEM of three independent experiments. One-way ANOVA was used for statistical analysis. Compared with the control group, *p < 0.05, **p < 0.01. **(C)** U251 cells were treated with 7 mM sodium butyrate for 48 h, followed by flow cytometry analysis to determine the percentage of Annexin V-positive apoptotic cells. **(D)** The mRNA levels of Survivin in U251 cells. **(E)** Results of the statistical analysis of Survivin protein levels in U251 cells. The data are presented as the means ± SDs of three independent replicates. Two-tailed, unpaired Student’s t test was used for statistical analysis. Compared with the control group, *p < 0.05, **p < 0.01.

To investigate the effect of sodium butyrate on the growth of glioma, U251 and GL261 glioma cells were exposed to various concentrations of sodium butyrate for 24, 48, 72 or 96 h. The viability of U251 and GL261 cells was determined using CCK-8 assay. As shown in **Figures 1A, B**, exposure to sodium butyrate led to a significant increase in the cytotoxicity toward these glioma cells in a dose- and time-dependent manner. Notably, U251 glioma cells were more sensitive to sodium butyrate than Gl261 cells. Subsequently, flow cytometry with Annexin V/PI staining was performed to examine whether sodium butyrate induces apoptosis in U251 cells. After 48 hours of exposure to sodium butyrate, the percentage of apoptotic (Annexin V-positive) U251 cells significantly increased (**Figure 1C**). As Survivin (also known as BIRC5) is known to play a crucial role in inhibiting tumor cell apoptosis (16), researchers have further examined the effect of sodium butyrate on Survivin. Consistent with the results for apoptosis, sodium butyrate treatment also led to a reduction in Survivin, as evidenced by decreased mRNA and protein expression of Survivin (**Figures 1D, E**) compared with that in the control group.

### 3.2 Treatment with sodium butyrate alters genome-wide gene expression in U251 cells

**Figure 2:**
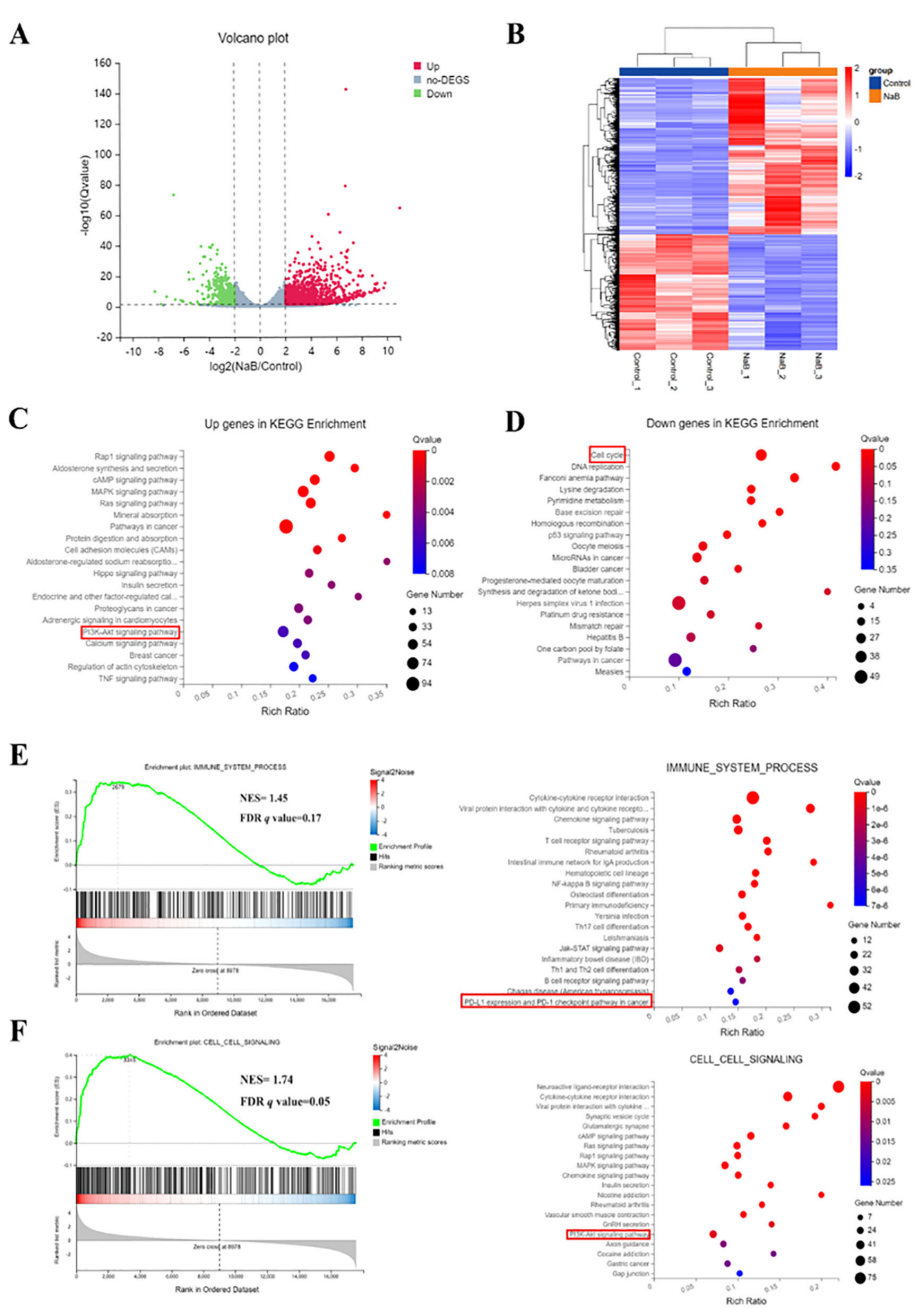
Treatment with sodium butyrate alters genome-wide gene expression in U251 cells. Volcano plot **(A)** and heatmap **(B)** of DEGs between control and NaB-treated U251 cells. **(C, D)** Bubble plot showing the enrichment of upregulated and downregulated genes. **(E, F)** Enrichment map of GSEA and KEGG pathways associated with upregulated and downregulated genes. NES, Normalized enrichment score; FDR, False positive detection rate. n=3.

To gain a deeper understanding of the mechanism underlying the treatment of glioma cells with the histone deacetylase inhibitor sodium butyrate, an RNA-seq assay was performed to profile genome-wide gene expression changes in U251 cells after treatment with sodium butyrate for 48 hours. After analyzing the data, differentially expressed genes (DEGs) between the control and sodium butyrate-treated (NaB) groups were identified based on the criteria of a |log2FC|≥2 and a Q value ≤ 0.05. Among these DEGs, 2022 were upregulated, and 1367 were downregulated (**Figures 2A, B**). KEGG analysis revealed that the most significantly altered pathways involved the cell cycle and DNA replication (**Figure 2C**). Additionally, significantly upregulated DEGs were enriched in the Rap1 signaling pathway, MAPK signaling pathway, and PI3K-AKT signaling pathway (**Figure 2D**).

Furthermore, the combined GSEA and KEGG analysis revealed that significantly downregulated or upregulated transcripts were involved in the immune system and cell signaling pathways, including PD-L1 expression, the PD-1 checkpoint pathway in cancer, and the PI3K-AKT signaling pathway (**Figures 2E, F**). Previous research has indicated that inducing Akt activity increases the protein expression of PD-L1, while inhibiting PI3K reduces PD-L1 protein expression in glioma cells (17). Therefore, the RNA-seq results suggest that sodium butyrate treatment affects a series of signaling pathways involved in cell cycle regulation and regulates the PI3K/AKT signaling pathway to impact the immune system of U251 cells.

### 3.3 Treatment with sodium butyrate blocks the U251 cell cycle

RNA-seq analysis revealed that NaB treatment significantly inhibited G2/M phase-related genes in U251 cells. The G2/M phase of the cell cycle is controlled by cell cycle protein-dependent kinases (CDKs) and cell cycle proteins of the cyclin family (18). CDC2, also known as CDK1, was the first cell cycle protein-dependent protein kinase discovered, and phosphorylated CDC2 plays a crucial role in cell cycle progression (19). CDC25C is a protease responsible for activating CDC2 through dephosphorylation and plays a key role in the regulation of mitosis (20). Cyclin B (CCNB) is essential for initiating mitosis at the G2/M checkpoint, while cyclin A (CCNA) is required for both the S and M phases of the cell cycle (21). Cyclin A2 (CCNA2) and cyclin B1 (cyclin B1, CCNB1) interact with CDC2 in cell cycle regulation, and the formation of the CDC2/cyclinB1 complex is necessary for transitioning cells from G2 to M phase (22).

**Figure 3:**
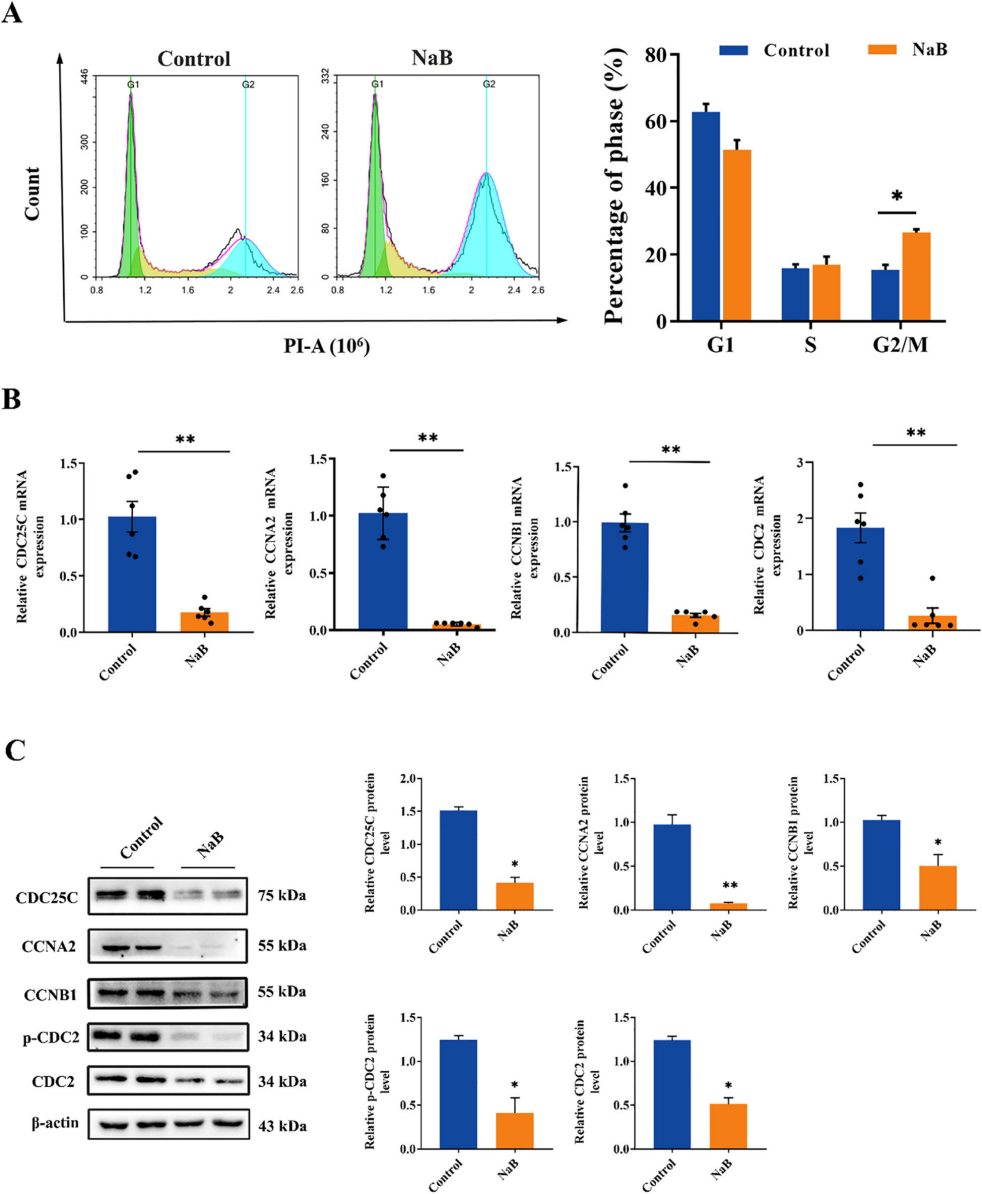
Treatment with sodium butyrate blocks the U251 cell cycle. **(A)** PI assay U251 cell cycle flow and statistical plot. **(B)** The mRNA levels of CDC25C, CCNA2, CCNB1 and CDC2 in U251 cells. **(C)** The protein levels of CDC25C, CCNA2, CCNB1, p-CDC2 and CDC2 in U251 cells. The data are expressed as the mean ± SEM of three independent experiments. Two-tailed, unpaired Student’s t test was used for statistical analysis. Compared with the control group, *p < 0.05, **p < 0.01.

To further verify the effect of NaB on the U251 cell cycle and related pathway proteins, we used PI staining to detect the cell cycle distribution of NaB-treated U251 cells. qPCR and Western blotting were used to detect the mRNA and protein levels of CDC2, CDC25C, CCNA2 and CCNB1, respectively, in U251 cells. The PI staining results demonstrated that NaB blocked U251 cells in the G2/M phase (**Figure 3A**, p < 0.05).

Furthermore, compared with those in the control group, the mRNA and protein levels of CDC2, CDC25C, CCNA2, and CCNB1 were significantly lower in the NaB-treated group (**Figures 3B, C**, p < 0.05), with the most significant reduction observed in the protein expression level of CCNA2 (**Figure 3C**, p < 0.01). These experimental results indicated that NaB significantly inhibited the cycle progression of U251 cells by downregulating the expression of cell cycle-related proteins and arresting them in the G2/M phase, which was consistent with the RNA-seq results.

### 3.4 Histone deacetylase expression upregulate in GBM clinical database and prolong glioma patients survival time

**Figure 4:**
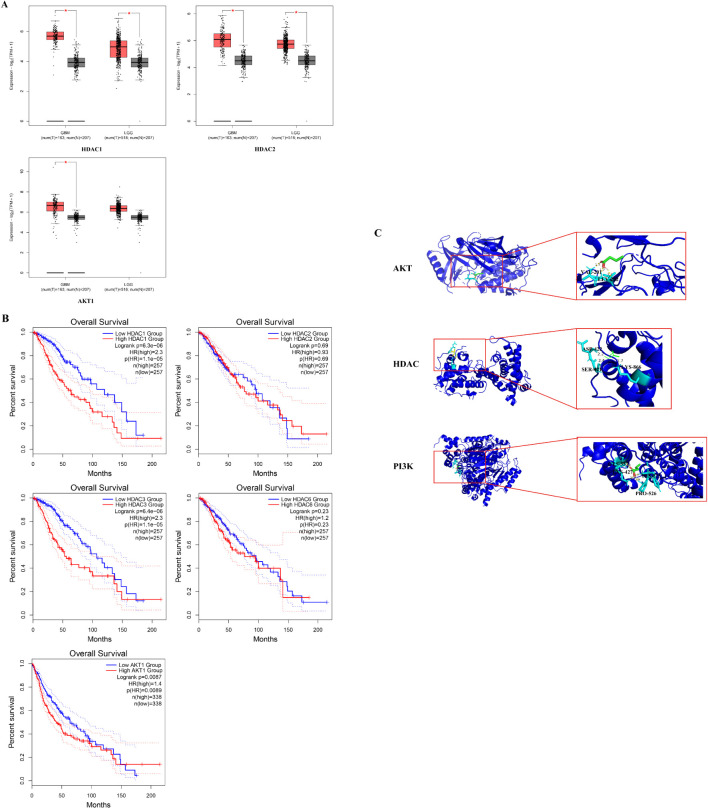
Histone Deacetylase expression upregulate in GBM clinical database and prolong glioma patients survival time. **(A)**The HDCA1, HDAC2 and AKT1 mRNA expression levels in common tumor tissues (*P < 0.05 *vs.* control group); **(B)** Overall survival of different HDAC1, HDAC2, HDAC3, HDAC6 and AKT1 level. The prognosis of the low HDAC1, HDAC3 and AKT1 expression groups was better than that of the high expression group. **(C)** The result diagram of molecular docking. The green 3D structural formula represents NaB; the blue bond represents the hydrogen bond at the binding site.

Besides the U251 cell RNA-seq assay results, we also search RNA-Seq data from the UALCAN database to explore the role of HDAC and PI3K/AKT genes in the context of glioma. The mRNA expression of HDAC1, HDAC2, HDAC3, HDAC6 and PI3K/AKT1 was analyzed in GBM and low grade glioma tissues. The mRNA expression levels of HDAC1, HDAC2 and AKT1 were relatively high in glioma (**Figure 4A**, p < 0.05), while those of HDAC3 and HDAC6 have no significant difference between glioma and normal tissues (**Supplementary Figure S1**, *p* > 0.05). The overall survival time of glioma patients with low HADC1, HDAC3 and AKT1 expression is better (**Figure 4B**, p < 0.05). Taken together, HDAC1 is considered as core gene in glioma.

According to the KEGG analysis, the critical pathway gene is AKT. Also, sodium butyrate act as HDACi in this study. Molecular docking showed a strong binding effect. The binding affinities of sodium butyrate to AKT (4EJN), PI3K (7jwz) and HDAC (7sme) were -4.13, -6.27 and -3.65 kcal/mol (**Figure 4C**). In addition, a coupling fraction of less than 0 kcal/mol indicates that the component can spontaneously bind to the target, less than -1.20 kcal/mol indicates a good affinity coupling, and less than -7 kcal/mol is considered as strong affinity coupling (2). The results indicated a good binding activity between NaB and the critical genes.

### 3.5 Sodium butyrate acts as a histone deacetylase inhibitor to upregulate PD-L1 protein levels in U251 cells through activation of the PI3K/AKT signaling pathway

**Figure 5:**
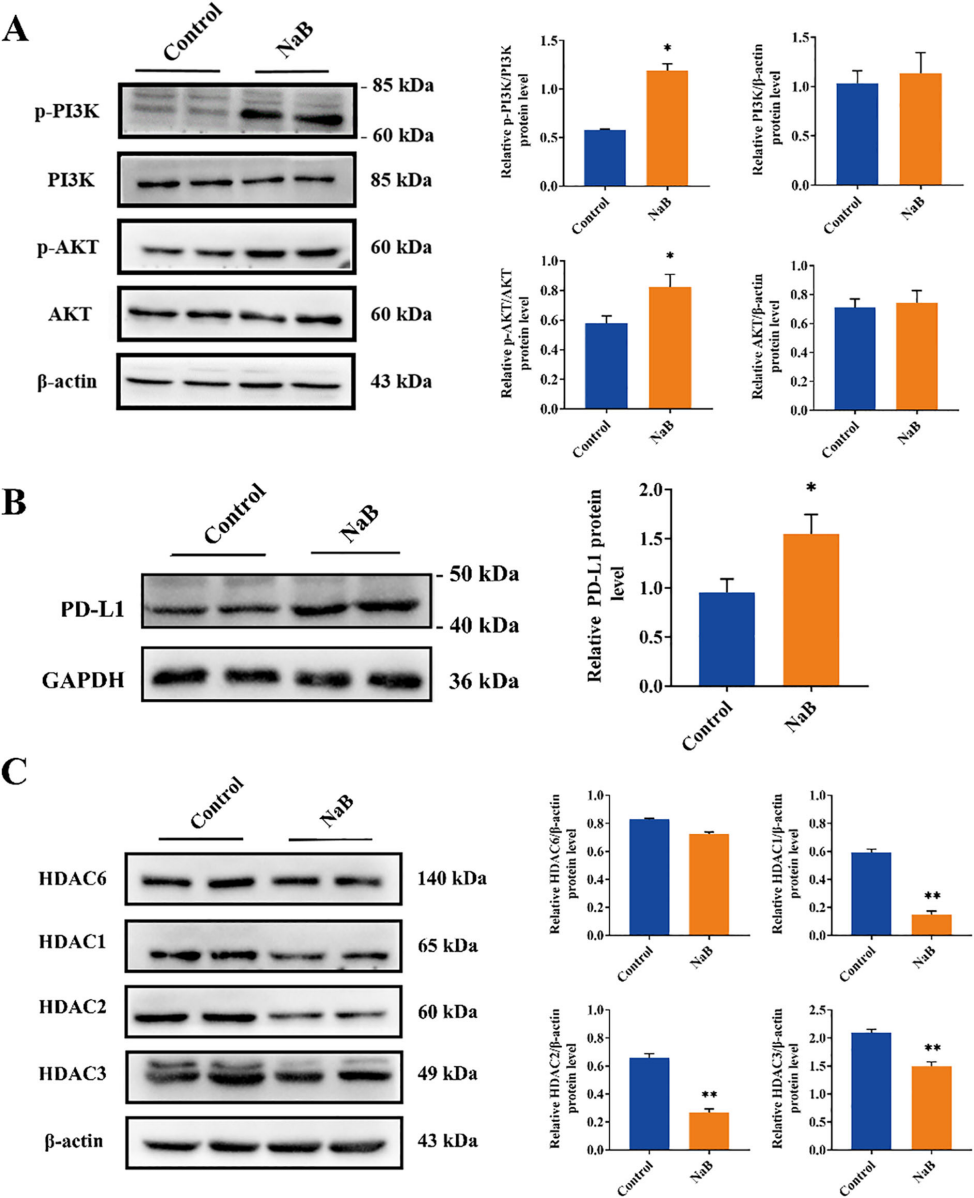
Sodium butyrate acts as a histone deacetylase inhibitor to upregulate PD-L1 protein levels in U251 cells through activation of the PI3K/AKT signaling pathway. **(A-C)** Representative immunoblots and quantitative analysis of the relative p-PI3K, PI3K, p-AKT, AKT, PD-L1, HDAC6, HDAC1, HDAC2, and HDAC3 protein expression in U251 cells. The data are expressed as the mean ± SEM of three independent experiments. Two-tailed, unpaired Student’s *t* test was used for statistical analysis. ^*^ *p* < 0.05, ^**^ *p* < 0.01, compared with the control group.

PD-1 and PD-L1 play crucial roles in suppressing immune responses and promoting self-tolerance by modulating T-cell activity, activating the apoptosis of antigen-specific T cells, and inhibiting the apoptosis of regulatory T cells (23). High levels of PD-L1 protein expression have been observed in most human cancers (24), including glioma (17). The expression of PD-L1 in the cancer microenvironment is regulated by multiple cytokines, such as IFN-γ (25) and IL-10 (26), and is also influenced by abnormal signaling pathways (27–29). The RNA-seq results in our study indicated that sodium butyrate treatment activated the PI3K/AKT signaling pathway in U251 cells (**Figure 2C**), and GSEA revealed that the differentially expressed genes were involved in PD-L1 expression and the PD-1 checkpoint pathway in cancer (**Figure 2E**). Based on these findings, we assessed the activation of the PI3K/AKT pathway and PD-L1 expression in U251 cells treated with sodium butyrate. As shown in **Figure 4A**, sodium butyrate treatment upregulated the protein levels of p-PI3K and p-AKT in U251 cells compared to those in the control group (*p* < 0.05). Additionally, sodium butyrate treatment significantly upregulated the protein level of PD-L1 in U251 cells (**Figure 5B**, p < 0.05). These results were consistent with the RNA-seq analysis.

Furthermore, HDAC inhibitors (HDACs) are known to induce apoptosis and cell cycle arrest, promote cell differentiation, and inhibit angiogenesis in tumor cells (30). Recent studies have shown that HDACs play important roles in tumor immune escape and drug resistance (31). Sodium butyrate, a class I HDAC inhibitor, has been widely used to study the role of histone acetylation in chromatin structure and function (32). Based on the RNA-seq results of U251 cells and glioma clinical data (**Figures 2**, **4**), we evaluated the expression levels of class I HDACs (HDAC1, 2, 3) and class IIb HDACs (HDAC6) in U251 cells treated with sodium butyrate. The results showed that sodium butyrate significantly inhibited the expression of HDAC1, HDAC2, and HDAC3 (**Figure 5C**, p < 0.01), while the HDAC6 protein level did not change significantly (**Figure 5C**, p > 0.05).

In conclusion, our findings suggest that sodium butyrate, a class I HDAC inhibitor, induces apoptosis and cell cycle arrest in U251 cells. Additionally, it activates the PI3K/AKT pathway, which in turn upregulates PD-L1 expression in U251 cells, potentially contributing to immunological changes in the tumor microenvironment.

### 3.6 Sodium butyrate enhanced the therapeutic effect of anti-PD-1 blockade in an orthotopic murine glioma model

**Figure 6:**
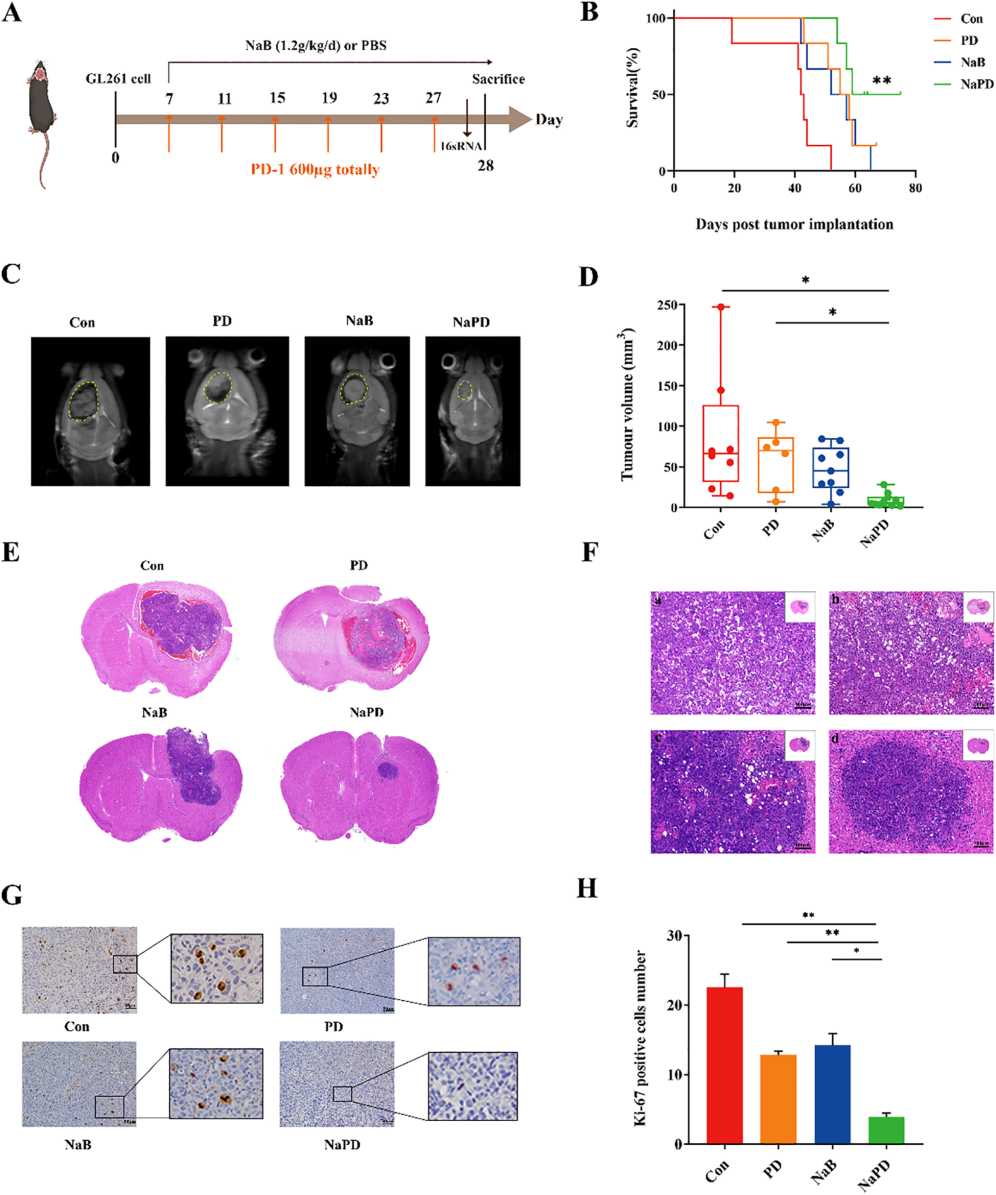
Combinatorial therapy with anti-mouse PD-1 mAb and sodium butyrate in an orthotopic murine glioma model. GL261 glioma cells were implanted into C57BL/6 mice. Seven days after inoculation, the mice were treated with NaB (1.2 g/kg/d), an anti-mouse PD-1 mAb (600 μg), a combination of the two reagents, or a solvent control as indicated **(A)**. K–M survival curves **(B)** were generated to evaluate glioma mouse survival time, n = 6, log-rank test. Compared with Con group, **p < 0.01, NS not show. The tumor volumes of the mice were measured with MRI after drug treatment for 21 days **(C)**, and the tumor volumes **(D)** were calculated. Compared with the Con and PD group, *p < 0.05, NS not show. **(E, F)** Tumor sections were prepared and stained with H&E for histological examination (×100). Moreover, representative Ki-67-stained sections (×200, **G**) and the numbers of Ki-67-positive cells in brain tissues **(H)** are shown. The experimental data are expressed as the mean ± SEM. Compared with the control and PD groups, **p < 0.01. Compared with the NaB group, *p < 0.05.

Recent preclinical studies have revealed that tumor PD-L1 expression reflects an immune-active microenvironment (33), and patients with tumors expressing PD-L1 are more likely to respond to anti-PD-1 therapy (34). GBM is considered a “cold tumor” characterized by a more immunosuppressive microenvironment, with poor T-cell infiltration, high bone marrow infiltration, and low PD-L1/PD-1 expression (35, 36). In this research, to investigate whether NaB could enhance the antitumor effect of anti-PD-1 therapy *in vivo*, we tested our hypothesis using GL261 cells implanted in mice. Seven days after the implantation of GL261 cells, the tumors were treated with sodium butyrate (1.2 g/kg/d), anti-mouse PD-1 mAb alone (600 μg per mouse), or a combination of both agents (**Figure 6A**). Survival of glioma mice was significantly prolonged by combination therapy (median survival, 67 days) compared with treatment with NaB or an anti-mouse PD-1 mAb alone (median survival, 54.5 and 56.5 days, respectively). Treatment with sodium butyrate alone did not affect tumor growth and failed to lead to tumor regression (0/6). Treatment with anti-mouse PD-1 mAb antibodies alone effectively controlled tumor growth, and 1 out of 6 tumors were eventually cleared. However, the combination of sodium butyrate with an anti-mouse PD-1 mAb substantially slowed tumor progression and resulted in 3 complete responses out of the 6 treated mice (**Figures 6B–F**). These findings suggested that NaB in combination with an anti-PD-L1 antibody enhanced the antitumor effect in an orthotopic murine glioma model. To examine whether the combination therapy regulated tumor proliferation, IHC staining for Ki-67 was performed. The number of Ki-67-positive cells was lower in the combination group than in the control group (**Figures 6G, H**, p < 0.01). To evaluate the safety of the combination therapy, we performed HE staining on the liver, kidneys, and Ileum and colon of the mice. HE results show that under long-term treatment, there were no morphological changes in the major metabolic organs of the glioma mice (**Supplementary Figure S3**). The administration of anti-PD1 in combination with sodium butyrate in glioma-bearing mice did not show significant metabolic toxicity, indicating a relatively high safety profile. In conclusion, the results of the *in vivo* study demonstrated that the combination of sodium butyrate with an anti-mouse PD-1 mAb had enhanced antitumor effects and increased survival in an orthotopic murine glioma model. This combination therapy could be a promising strategy to overcome the immunosuppressive microenvironment of glioblastoma and improve the response to anti-PD-1 therapy.

### 3.7 Combination therapy increases immune cytokines and T-cell infiltration in mice bearing established intracranial gliomas

**Figure 7:**
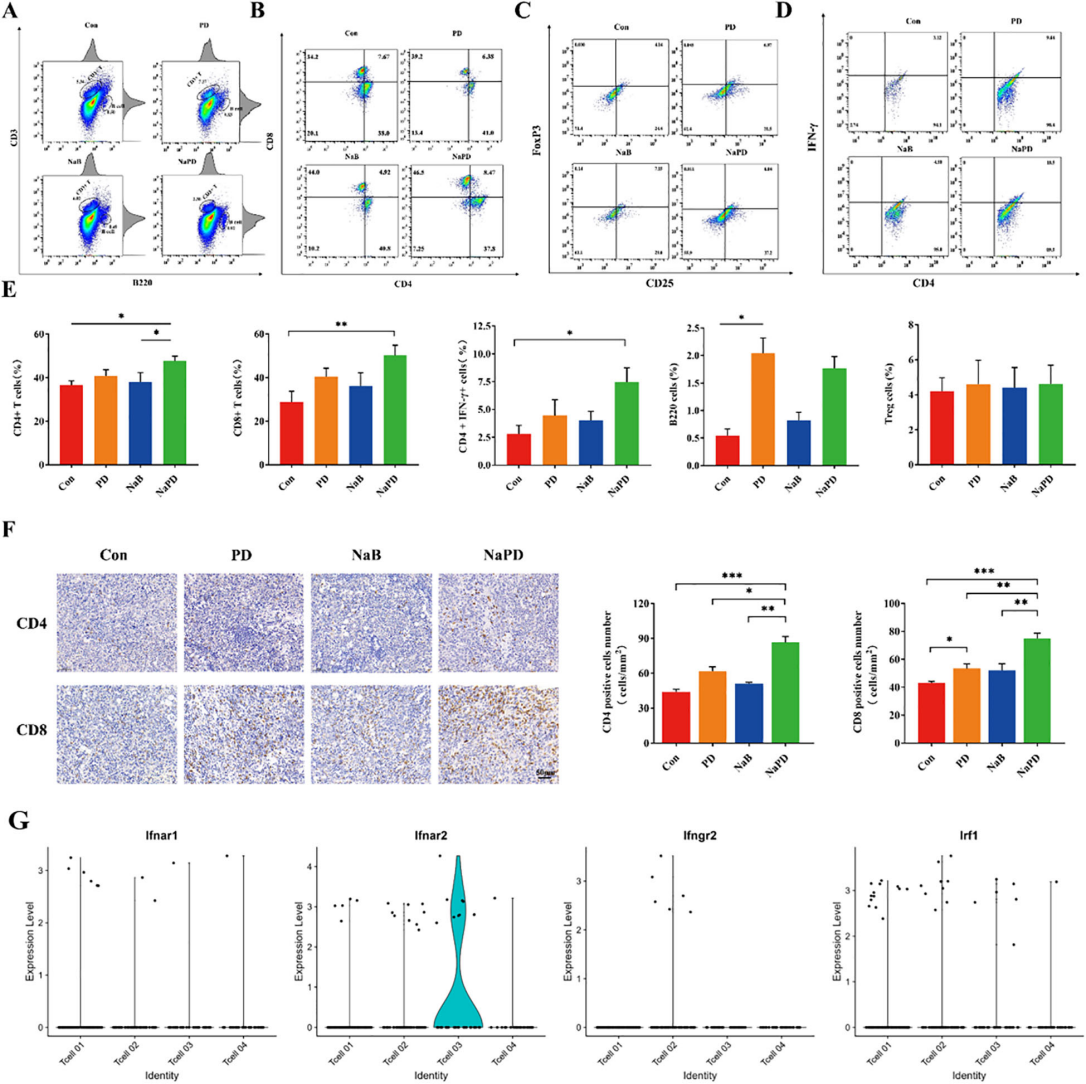
Anti-mouse PD-1 mAb and sodium butyrate combination treatment retards tumor growth by increasing T-cell infiltration. **(A, B)** After treatment, different groups of tumors were subjected to FACS to determine the percentage of CD4^+^ and CD8^+^ cells among the total viable cells. Compared with the control treatment, combination therapy dramatically increased the percentages of CD4^+^ and CD8^+^ tumor-infiltrated cells. **(C)** Treg cells did not significantly change among the groups. **(D)** IFN-γ production in CD4^+^ TILs in each group. Combination treatment increased IFN-γ production in CD4^+^ TILs. **(E)** The levels of CD4^+^, CD8^+^ T, Treg, CD4^+^ IFN-γ and B cells in tumor tissues from each group. **(F)** Immunohistochemical analysis of CD4 and CD8 in tumor tissue. **(G)** Violinplots shows ifnar2 gene has higher scores in IFN-γ related genes. The experimental data are expressed as the mean ± SEM, *p<0.05. **p<0.01. ***p<0.001.

We performed single-cell transcriptomic analysis on the orthotopic glioma mouse model (GSE246154) from the GEO database using R. The results indicated that the Ifnar2 gene was enriched in T cells (**Supplementary Figures S4G, H**), while the CD68 and CD33 genes were less enriched in macrophages (**Supplementary Figure S5**). T cell infiltration in the mouse glioma cells was low (**Supplementary Figure S4A**), further validating that glioma is a ‘cold’ tumor. The percentage of tumor-infiltrating T lymphocytes (TILs) in the four groups was assessed using flow cytometry (**Figure 7A**). Compared with those in the other groups, the proportions of CD3^+^CD8^+^ and CD3^+^CD4^+^ cells in the combination therapy group were significantly greater (**Figure 7B**, for the CD4^+^ T cell proportion, the control group had 36.58% ± 4.48%, the PD group had 40.80% ± 6.34%, the NaB group had 37.96% ± 9.84%, and the combination group had 47.66% ± 4.94%; For the CD8^+^ T cell proportion, the control group had 28.82% ± 11.07%, the PD group had 40.44% ± 8.67%, the NaB group had 36.08% ± 13.77%, and the combination group had 50.11% ± 10.53%). However, the percentage of Treg cells did not significantly differ between the treatment groups and the control groups (**Figure 7C**, p>0.05). To further investigate the immune response, TILs were cultured for 4-6 hours, and the production of IFN-γ in CD4^+^ TILs was examined (**Figure 7D**). Compared with the control treatment, the combination treatment significantly increased the production of IFN-γ by TILs (**Figure 7E**, *p*<0.05). In addition, CD4 and CD8 positive cells were characterized in the tumor tissue using immunohistochemical analysis. The combination therapy group exhibited a substantial increase in the number of CD8 and CD4 positive cells (**Figure 7F**, *p*<0.001), which was consistent with the flow cytometry results. Moreover, the CD8^+^ T-cell number was also significantly greater in the anti-mouse PD-1 group than in the control group (**Figure 7G**, *p*<0.05). Overall, the results indicate that the combination of sodium butyrate with an anti-mouse PD-1 antibody effectively retards tumor growth and enhances the antitumor response by promoting and optimizing the tumor microenvironment. This combination therapy appears to induce a more immune-active microenvironment, with increased TILs, elevated CD4^+^ and CD8^+^ T-cell ratios, and enhanced IFN-γ production, ultimately contributing to the antitumor effects observed.

### 3.8 Effects of sodium butyrate and PD-1 inhibitor administration on the gut microbiota and its metabolites in glioma mice

**Figure 8:**
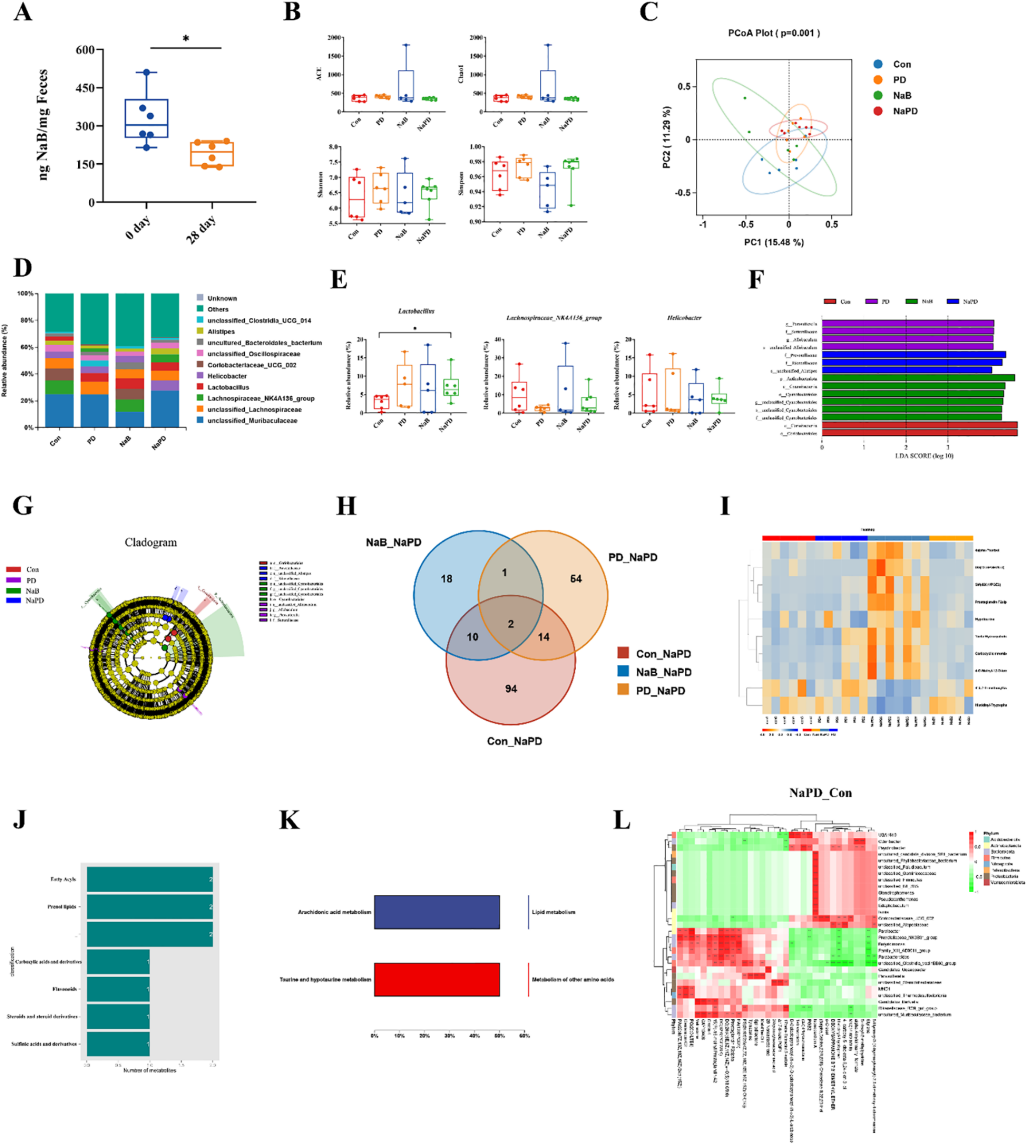
Combined analysis of the gut microbiota and its metabolites in glioma mice. **(A)** NaB content in the feces of mice before and after glioma implantation. The data are expressed as the mean ± SD, n = 6. *p < 0.05. Changes in the gut microbiota diversity of different groups, **(B)** ACE, Chao1, Shannon and Simpson indices. **(C)** PCoA analysis of gut microbiota β diversity. **(D)** Species distribution map of the gut microbiota at the genus level. **(E)** Relative abundances of *Lactobacillus*, *Lachnospiraceae_NK4A136_group* and *Helicobacter*. The experimental data are expressed as 5 - 95% confidence intervals, n = 5-6. Compared with the model group. *p < 0.05. **(F, G)** LEfSe analysis of the gut microbiota in different groups. **(H)** Venn diagram of the differentially abundant metabolites between the NaPD and Con groups and between the PD group and the NaB group. **(I-K)** Classification of differentially abundant metabolites in the combination group and their functional analysis. **(L)** Differentially abundant metabolite clustering-microbial correlation analysis for the combination group (genus level).

Notably, glioma growth has been reported to induce changes in fecal SFCA levels and neurotransmitters (37, 38). In the present study, LC-MS/MS analysis revealed a significant decrease in fecal sodium butyrate content in tumor-bearing mice compared to pre-tumor implantation levels (**Figure 8A**).

We also examined changes in the gut microbiota composition among the four treatment groups using 16S rRNA sequencing. The α-diversity of the gut microbiota was assessed using four different metrics. The results showed that the combination therapy of sodium butyrate with anti-mouse PD-1 did not significantly affect the α-diversity of the gut microbiota in glioma-bearing mice (**Figure 8B**). However, β diversity analysis (PCoA) demonstrated that the gut microbiota in the combination group was more similar to that of the anti-mouse PD-1 group, and more distinct from that of the NaB group (**Figure 8C**). The gut microbiota in the four groups was dominated by *Bacteroidetes* and *Firmicutes* at the phylum level, and significant changes in each group were not observed (data not shown). Interestingly, genus-level analysis revealed that the top-ranked genera were *Lachnospiraceae_NK4A136_group*, *Lactobacillus* and *Helicobacter* (**Figure 8D**). *Lactobacillus* is a recognized probiotic and is often used as a fermenting agent in yogurt (39). Compared to that in the control group, the relative abundance of *Lactobacillus* was significantly greater in the combination group (*p* < 0.05). *Lachnospiraceae_NK4A136_group* was one of the genera that produces SCFAs (40); however, there was no difference among the groups of mice (*p* > 0.05), indicating that exogenous administration of NaB had no significant effect on the abundance of SCFA-producing microbiota. *Helicobacter* is a pathogenic bacteria (41), and the relative abundance of *Helicobacter* did not differ significantly among the four groups of mice (*p* > 0.05), suggesting that *Helicobacter* abundance was not significantly associated with glioma treatment (**Figure 8E**). In the combination group, *Bacteroides* was enriched (*f:Rikenellaceae*, *f:Prevotellaceae*, *s:unclassified_Alistipes*, **Figures 8F, G**).

The untargeted metabolomics analysis by GC–MS revealed 10 shared differentially abundant metabolites in the combination group compared to the other three groups, suggesting their specificity in the combination group (**Figure 8H**). We annotated the 10 shared differentially abundant metabolites, and the results are shown in **Figure 8I**. The shared differentially abundant metabolites in the combination group were fatty acyls, prenol lipids, carboxylic acids and derivatives, flavonoids, steroids and steroid derivatives, and sulfinic acids and derivatives (**Figure 8J**). To investigate the function of the differentially abundant metabolites in the combination group, we performed KEGG enrichment analysis, and the results showed that the differentially abundant metabolites were mainly involved in lipid metabolism and other amino acid metabolism (**Figure 8K**).

Further correlation analysis was performed between the differentially abundant metabolites and the differential flora at the genus level. The heatmap shows the magnitude and direction of correlation between various differentially abundant metabolites and each differential flora classification (**Figure 8L**). Significant correlations (*p*<0.05) between metabolite clusters and microorganisms were observed, indicating potential interactions between specific gut microbial communities and metabolites in the combination group.

These results collectively indicate that the combination of sodium butyrate with an anti-mouse PD-1 antibody affects the gut microbiota composition and metabolic profiles in glioma-bearing mice. The alterations in the gut microbiota and associated metabolites might contribute to the observed enhanced antitumor effects and improved tumor microenvironment in response to the combination therapy.

## 4 Discussion

In our work, we experimentally investigated the role of sodium butyrate in glioma cells and its combination with a PD-1 inhibitor in a glioma mouse model. Sodium butyrate, as one of the gut microbiota metabolites, upregulates PD-L1 expression in glioma cells by regulating the PI3K/AKT pathway, helping to transform GBM cells from a “cold tumor” to a “hot tumor”, and improving the efficacy of PD-1 inhibitors for GBM treatment. Single-cell data analysis indicates that glioma-bearing mice have a low proportion of T cells, classifying glioma as a “cold tumor.” Additionally, IFNγ related genes enrichment are minimal. The combination therapy promoted tumor immune cell infiltration, prolonged the survival time of glioma-bearing mice and inhibited glioma growth. Since sodium butyrate (NaB) is a nontoxic four-carbon short-chain fatty acid produced during the fermentation of dietary fiber in the colon and is a natural inhibitor of histone deacetylase, we demonstrated that glioma growth decreases the NaB content in the feces. Furthermore, combination therapy increases the proportion of probiotics in the gut microbiota of tumor-bearing mice, promotes antitumor metabolites and decreases metabolites that affect immune cell function, thus inhibiting the growth of gliomas in mice through the “brain-gut axis”.

Histone acetylations are a key component of epigenetics, and inhibition of histone deacetylases induces acute hyperacetylation of epigenetic regulators at the histone-chromatin interface, resulting in the regulation of RNA polymerase II (Pol II)-driven transcription (42). In cancer cells, this regulation of transcription includes tumor suppression, antigen processing and expression mechanisms, and re-expression of tumor antigen-associated genes (30, 43). NaB is a natural histone deacetylase inhibitor (44). To investigate the effect of NaB on glioma cells, we first examined the proliferation of NaB-treated U251 cells using a CCK-8 assay, and the results showed that treatment of glioma cells with 7 mM NaB for 48 h significantly inhibited glioma cell proliferation. Second, Annexin V/PI double staining showed that NaB significantly induced apoptosis in glioma cells. qPCR and WB results showed that NaB downregulated Survivin protein levels in glioma cells. Finally, PI staining flow cytometry, qPCR and WB showed that NaB blocked the cell cycle of glioma cells by downregulating CDC2, CDC25C, CCNA2 and CCNB cell cycle-related proteins. The above results suggest that NaB, an HDACi, can inhibit the growth of glioma cells.

Both abnormally upregulated PD-L1 expression and a lack of PD-L1 led to ineffective PD-1/PD-L1 inhibitors. However, the expression of PD-L1 or PD-1 is a prerequisite for therapeutic efficacy. In prostate cancer, low PD-L1 expression is associated with a poorer PD-1 blockade response (24). We used RNA-seq to detect DEGs in NaB-treated U251 cells. KEGG enrichment analysis revealed that the upregulated genes were enriched mainly in cancer-related signaling pathways, and GSEA further revealed that the upregulated genes were involved in immune-related systems. GSEA further revealed that the upregulated genes were enriched mainly in PD-1/PD-L1 immune checkpoints. To verify the accuracy of the sequencing results, we examined the PD-L1 protein levels in NaB-treated U251 cells by Western blot and found that NaB significantly upregulated PD-L1 protein expression in U251 cells compared to that in the control group, which was consistent with the transcriptome sequencing results.

PD-L1 transcript levels are regulated by multiple signaling pathways, and our differential gene set enrichment analysis (GSEA) revealed that among the signaling pathway-related gene clusters, upregulated genes were enriched in the PI3K/AKT pathway. The next experiment also demonstrated that NaB activates the PI3K/AKT pathway in glioma cells, as evidenced by the significant upregulation of p-PI3K and p-AKT (**Figure 5A**). Activation of the PI3K pathway has been reported to lead to increased PD-L1 expression in glioma cells (17). IFNγ mediates the activation of the AKT-mTOR signaling pathway in non-small cell lung cancer, increasing its PD-L1 expression (45). Similarly, activation of the MAPK and PI3K pathways in melanoma also upregulates PD-L1 expression (46). This finding is consistent with our experimental results, both suggesting that the PI3K/AKT signaling pathway plays an important role in regulating PD-L1 transcript levels. Furthermore, there are other signaling pathways that regulate PD-L1 expression, such as in lung cancer cells, where PD-L1 expression may be regulated by the epidermal growth factor receptor (EGFR) pathway (47). In T-cell lymphoma, STAT3 silencing reduces PD-L1 expression (48). In breast cancer, reactive oxygen species (ROS) accumulation activates the downstream NF-κB signaling pathway, which increases PD-L1 protein levels (49). However, whether NaB mediates PD-L1 expression by regulating other signaling pathways and how NaB, as an HDACi, activates the PI3K/AKT signaling pathway in glioma cells in terms of epistasis modification need further investigation.

Since NaB is mainly metabolized by the gut microbiota *in vivo*, we performed a combined analysis of microorganisms and their metabolites in the feces of each group of mice. 16S RNA sequencing revealed no significant differences between the different groups at the phylum level and no significant changes in the *Firmicutes*, *Bacteroidetes* or F/B ratio. At the genus level, the abundance of *Lactobacillus* was significantly greater in the combination group than in the control group. *Lactobacillus* is a recognized probiotic and has potential anticancer effects. *Lactobacillus* can interact with proteins that regulate the cell cycle and inhibit the proliferation of cancer cells (39). Researchers found that a gavage mixture of *Lactobacillus* and *Bifidobacterium* inhibited the growth of brain gliomas in mice (50). We therefore hypothesized that the combination therapy was able to increase the abundance of probiotics in the gut microbiota of mice to promote the therapeutic effect on glioma. *Lachnospiraceae_NK4A136_group* is a genus that produces SCFAs. However, there was no difference among the groups of mice (*p*>0.05), indicating that exogenous administration of NaB had no significant effect on the abundance of SCFA-producing microbiota. The effect of the gut microbiota on the organism acts mainly through its metabolites, and we used LC-MS to measure the changes in metabolites of the gut microbiota in each group. We found that the top three upregulated metabolites that differed between the combination and control groups were statins, ethyladipic acid and N-methylphenylethanolamine. The three most downregulated metabolites were 4-O-methylgalactinol, 2-isopropyl-1,4-hexadiene and methyl linoleate. In addition to their cholesterol-lowering effects (51), statins have proapoptotic, antiangiogenic, and immunomodulatory effects. Statins have been shown to inhibit the growth of several cancer cell types, such as neuroblastoma, breast cancer, melanoma, and acute myeloid leukemia cells (52). Therefore, we speculate that the better therapeutic effect of NaB in combination with a PD-1 inhibitor in glioma may be related to the increase in active antitumor substances in the metabolites of the gut microbiota and the decrease in substances that inhibit the function of T cells.

Taken together, our study demonstrates that glioma growth reduces the levels of microbiome-derived sodium butyrate in mice. Sodium butyrate inhibits glioma cell growth *in vitro*, arrests the cell cycle, and enhances PD-L1 expression in glioma cells by activating the PI3K/AKT pathway, which may facilitate the conversion of gliomas from ‘cold’ tumors to ‘hot’ tumors. In an orthotopic glioma mouse model, sodium butyrate enhances the efficacy of anti-PD-1 immunotherapy by modulating the immune infiltration of cytotoxic CD8^+^ T cells. These findings suggest that sodium butyrate is a promising biomarker that could significantly improve the therapeutic outcomes for glioma patients.

## 5 Conclusions

First, sodium butyrate significantly inhibited the viability of U251 and GL261 glioma cells, with U251 cells showing higher sensitivity. Flow cytometry indicated an increased percentage of apoptotic U251 cells after 48 hours of NaB treatment. NaB also reduced survivin mRNA and protein levels in U251 cells.

Besides, RNA-seq analysis revealed significant changes in gene expression related to the cell cycle, PD-L1 expression, and the PI3K/Akt pathway after NaB treatment. NaB downregulated CDC2 and other cell cycle-related proteins, activated the PI3K/AKT pathway, and increased PD-L1 expression on the cell surface. NaB also inhibited HDAC1 expression. Moreover, analysis of a glioblastoma multiforme (GBM) clinical database indicated that lower expressions of HDAC1, HDAC3, and AKT1 correlated with better overall survival in glioma patients. Molecular docking studies showed that NaB could bind well to AKT1, PI3K, and HDAC proteins. In a mouse glioma model, the combination of NaB and a PD-1 inhibitor significantly reduced tumor size and extended survival. This combination therapy increased immune cytokines and T-cell infiltration in glioma-bearing mice. Last, NaB also act as a major metabolite of the gut microbiota, altered the gut microbiota composition and metabolic profiles in glioma mice, enhancing the antitumor effects and improving the tumor microenvironment.

Overall, this study is the first to discover that the combination of sodium butyrate with an anti-PD-1 antibody demonstrates significant potential in treating gliomas by modulating immune responses and affecting gut microbiota, providing insights into overcoming the limitations of current glioma clinical treatments.

## Data Availability

The data presented in the study are deposited in the NCBI repository, accession number PRJNA1243548 and PRJNA1243480.

## Glossary

ALT: Alanine aminotransferase
AST: Aspartate aminotransferase
BUN: Blood urea nitrogen
CCNA2: Cyclin A2
CCNB1: Cyclin B12
CDK: Cyclin-dependent kinases
CRE: Creatinine
CTLA-4: Cytotoxic T-lymphocyte-associated protein 4
DAB: 3,3’-diaminobenzidine
DEPC: Diethylpyrocarbonate
DMEM: Dulbecco’s modified eagle medium
ECL: Enhanced chemiluminescence
ES: Enrichment score
FDA: Food and drug administration
GC-MS: Gas chromatograph-mass spectrometry
GFAP: Glial fibrillary acidic protein
GBM: Glioblastoma
GO: Gene ontology
GSEA: Gene Set Enrichment Analysis
HDAC: Histone deacetylase
HGG: High-grade glioma
HMDB: Human metabolome database
ICI: Immune checkpoint inhibitors
IFN: Interferon
IHC: Immunohistochemistry
JAK: Janus kinase
KEGG: Kyoto encyclopedia of genes and genomes
LC-MS: Liquid chromatograph-mass spectrometry
LEfSe: Linear discriminant analysis Effect Size
lncRNAs: long noncoding RNA
MRI: Magnetic resonance imaging
NaB: Sodium butyrate
OS: Overall Survival
PAGE: Polyacrulamide gel electrophoresis
PBS: Phosphate buffered saline
PD-1: Programmed cell death-1
PD-L1: Programmed cell death 1 ligand 1
PFS: progression-free survival
PI: Propidium
PI3K: Phosphatidylinositol 3-kinase
PMSF: Phenylmethanesulfonyl fluoride
Pol II: RNA polymerase II
PVDF: Polyvinylidene fluoride
qPCR: Real-time quantitative polymerase chain reaction
SCFA: Short chain fatty acid
SDS: Sodium dodecyl sulfate
SRS: Stereotactic radiosurgery
STAT3: Signal transducer and activator of transcription
T2WI: T2 weighted imaging
TIM-3: T cell immunoglobulin-3
TILs: Tumor-infiltrating lymphocytes
TME: Tumor microenvironment
TMZ: Temozolomide
Treg: Regulatory T-cells
TTF: tumor treating fields
VIP: Variable importance in the projection
WHO: World health organization
