# Supplementary material for: Combination of Sodium Butyrate and Immunotherapy in Glioma: regulation of immunologically hot and cold tumors via gut microbiota and metabolites
Source: Front Immunol. 2025 Apr 14;16:1532528. doi: 10.3389/fimmu.2025.1532528 (PMC12035444; doi:10.3389/fimmu.2025.1532528)
Supplement: Supplementary file 1 [file DataSheet1.docx]

Supplementary Material

# Supplementary Figures and Tables

## Supplementary Tables

| Gene | Forword（5’-3’） | Reverse（5’-3’） |
| --- | --- | --- |
| CCNA2 | CGCTGGCGGTACTGAAGTC | GAGGAACGGTGACATGCTAT |
| CCNB1 | AATAAGGCGAAGATCAACATGGC | TTTGTTACCAATGTCCCCAAGAG |
| CDC25C | TCTACGGAACTCTTCTCATCCAC | TCCAGGAGCAGGTTTAACATTTT |
| CDC2 | AAACTACAGGTCAAGTGGTAGCC | TCCTGCATAAGCACATCCTGA |
| TNF-α | TATGGCTCAGGGTCCAACTC | GCTCCAGTGAATTCGGAAAG |
| IFN-γ | AATAAGGCGAAGATCAACATGGC | TTTGTTACCAATGTCCCCAAGAG |
| IL-10 | ATGCAGGACTTTAAGGGTTACTTG | TAGACACCTTGGTCTTGGAGCTT |
| IL-6 | ACAAGTCGGAGGCTTAATTACACAT | TTGCCATTGCACAACTCTTTTC |
| GAPDH | AGCGAGACCCCACTAACATC | GGTTCACACCCATCACAAAC |

**Table Supplementary 1.** PCR Primer Sequences

## Supplementary Figures

**
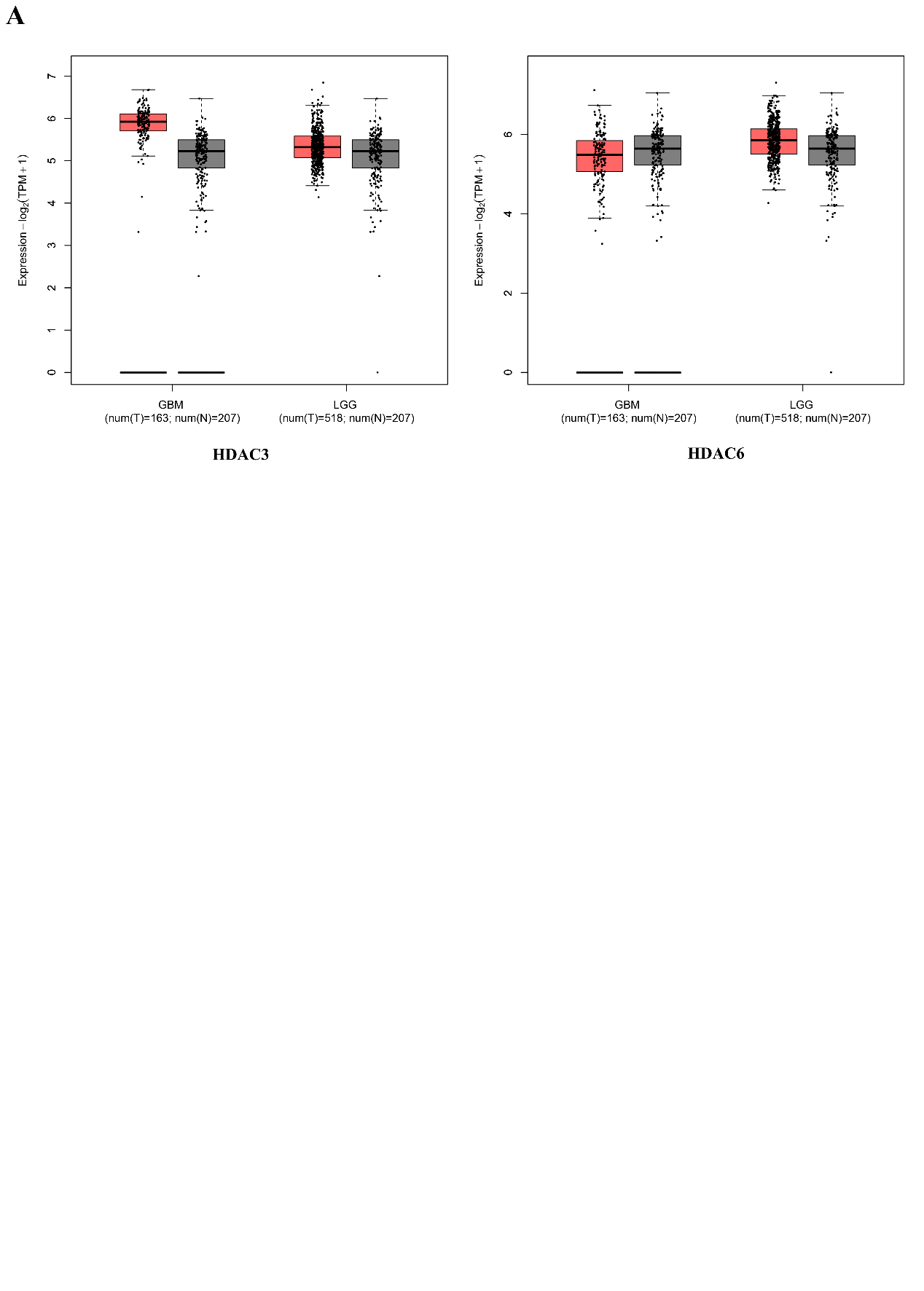
**

**Supplementary Figure 1.** Histone Deacetylase expression upregulate in GBM clinical database and prolong glioma patients survival time. (A)The HDCA3 and HDAC6 mRNA expression levels in common tumor tissues (*P > 0.05 vs. control group).


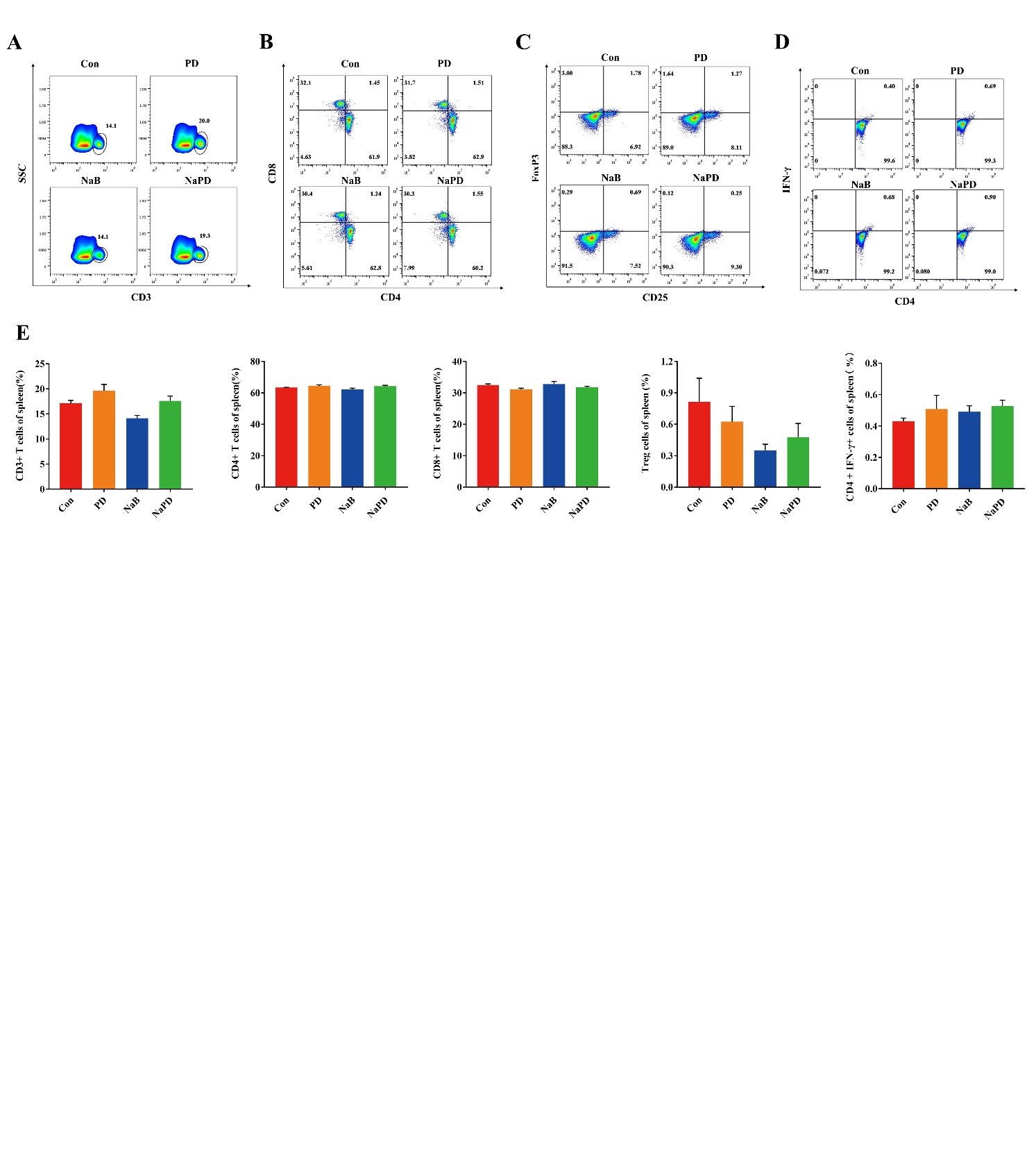


**Supplementary Figure 2.** Immune cytokines and T cell infiltration of glioma bearing mice spleen have no significant change. (A-E) The levels of, CD4+, CD8+T, Treg, CD4+ IFN-γ and B cells in spleen in each group. Experimental data are expressed as mean ± SEM.


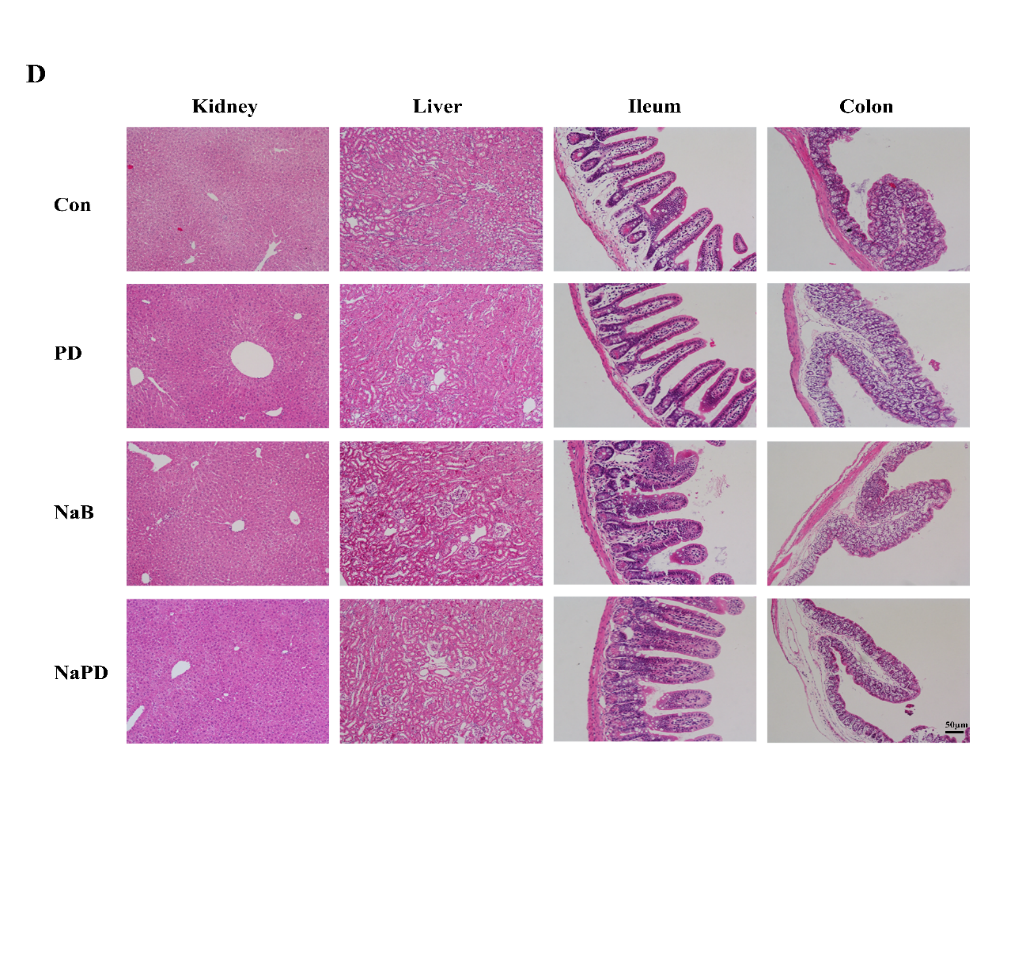

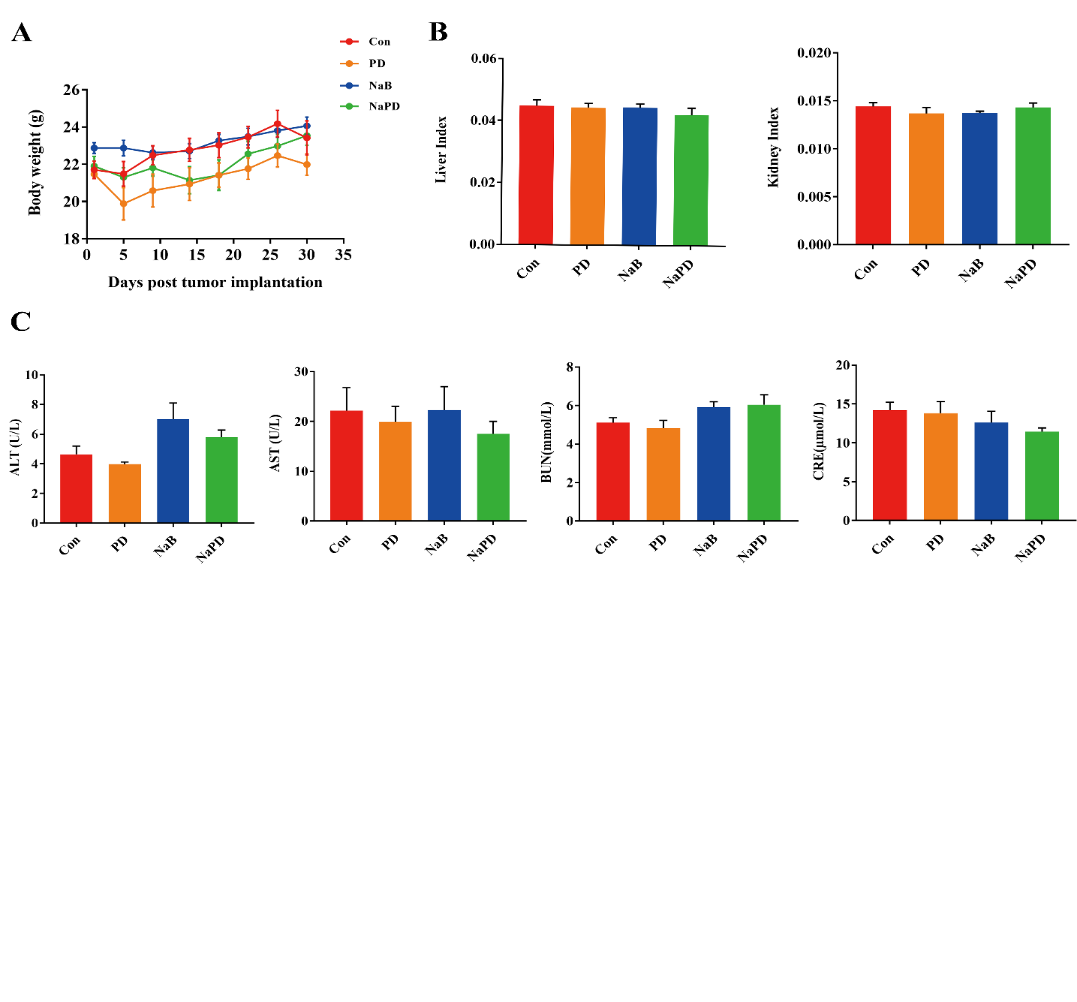


**Supplementary Figure 3. Safety evaluation of PD-1 inhibitor and NaB in glioma mice.** (A) Changes of body weight in each group mice. (B) Quantitative analysis on liver and kidney index in each group. (C) Quantitative analysis on liver and kidnry function biomarkers (ALT, AST, BUN and CRE). (D) H&E staining of the liver, kidneys, Ileum and colon collected from each group mice. n=6. Experimental data are expressed as mean ± SEM.


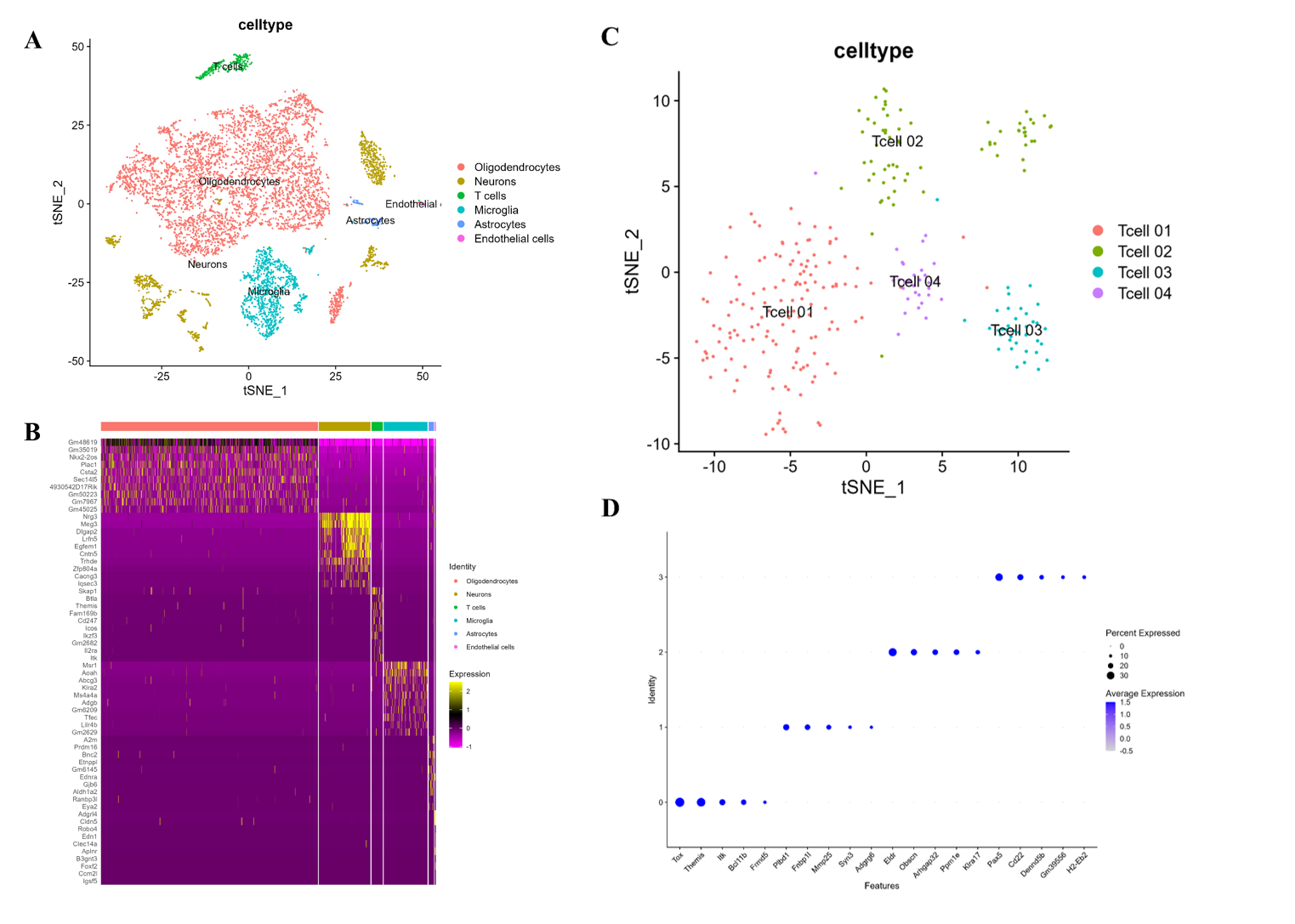


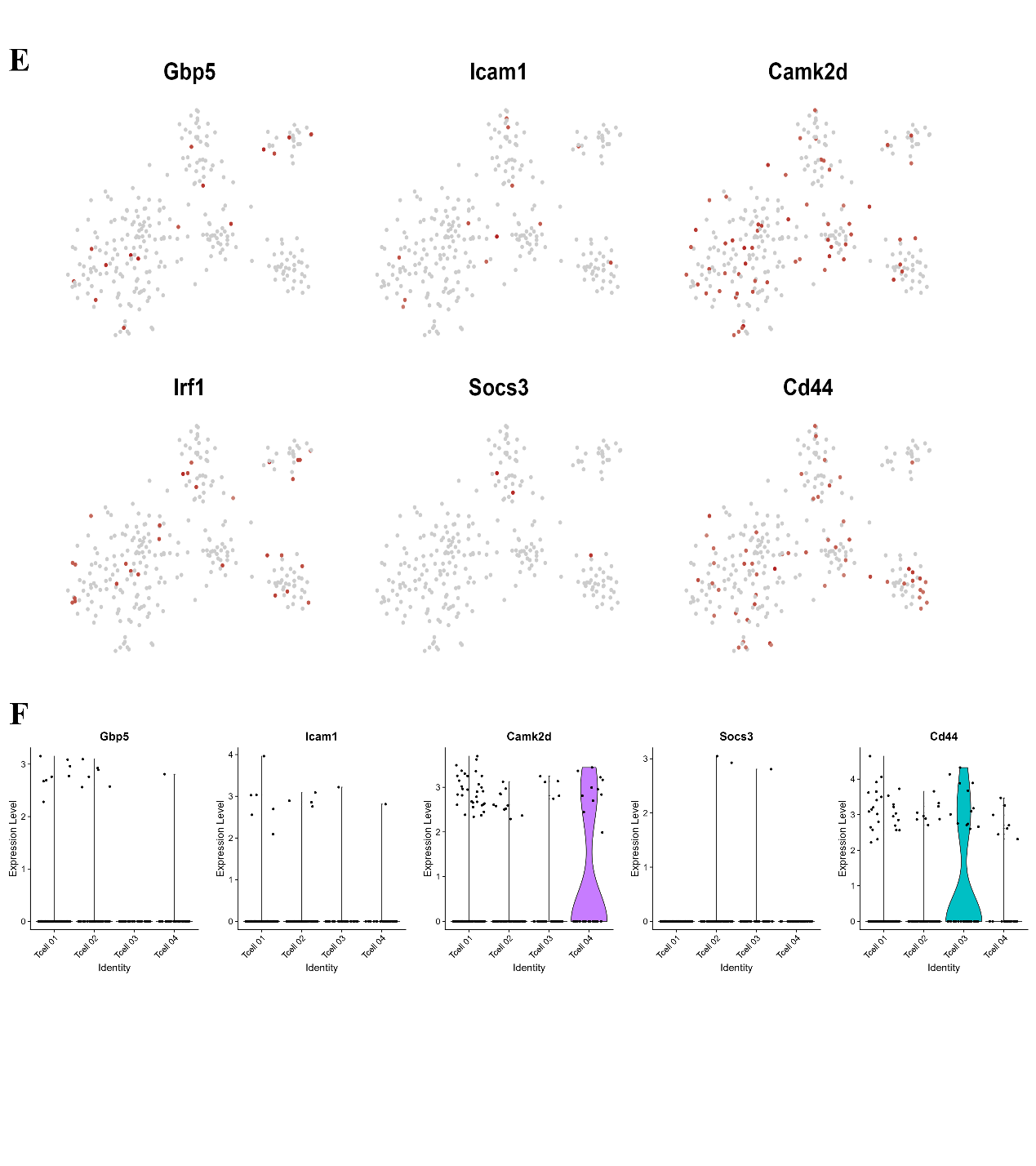


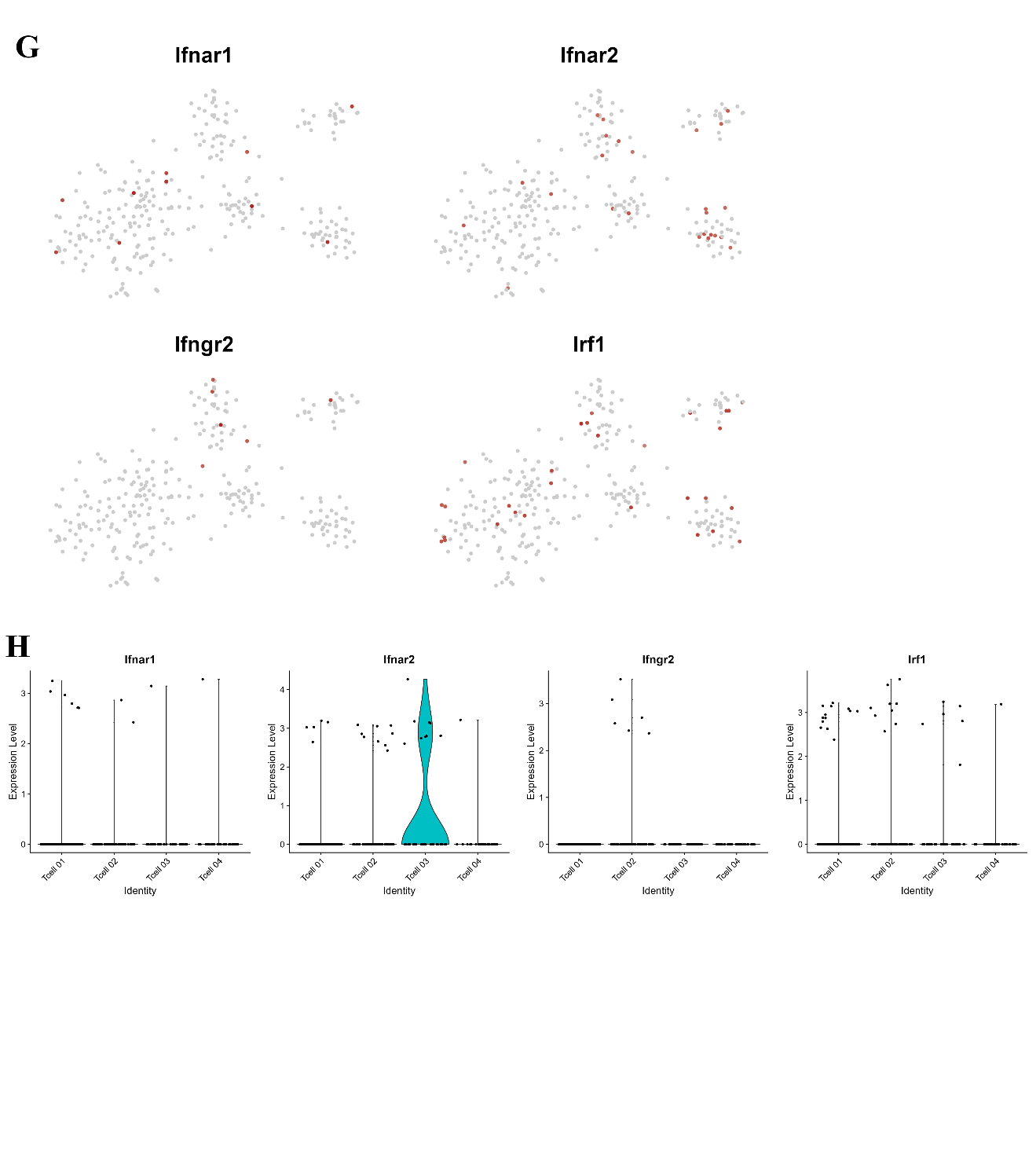

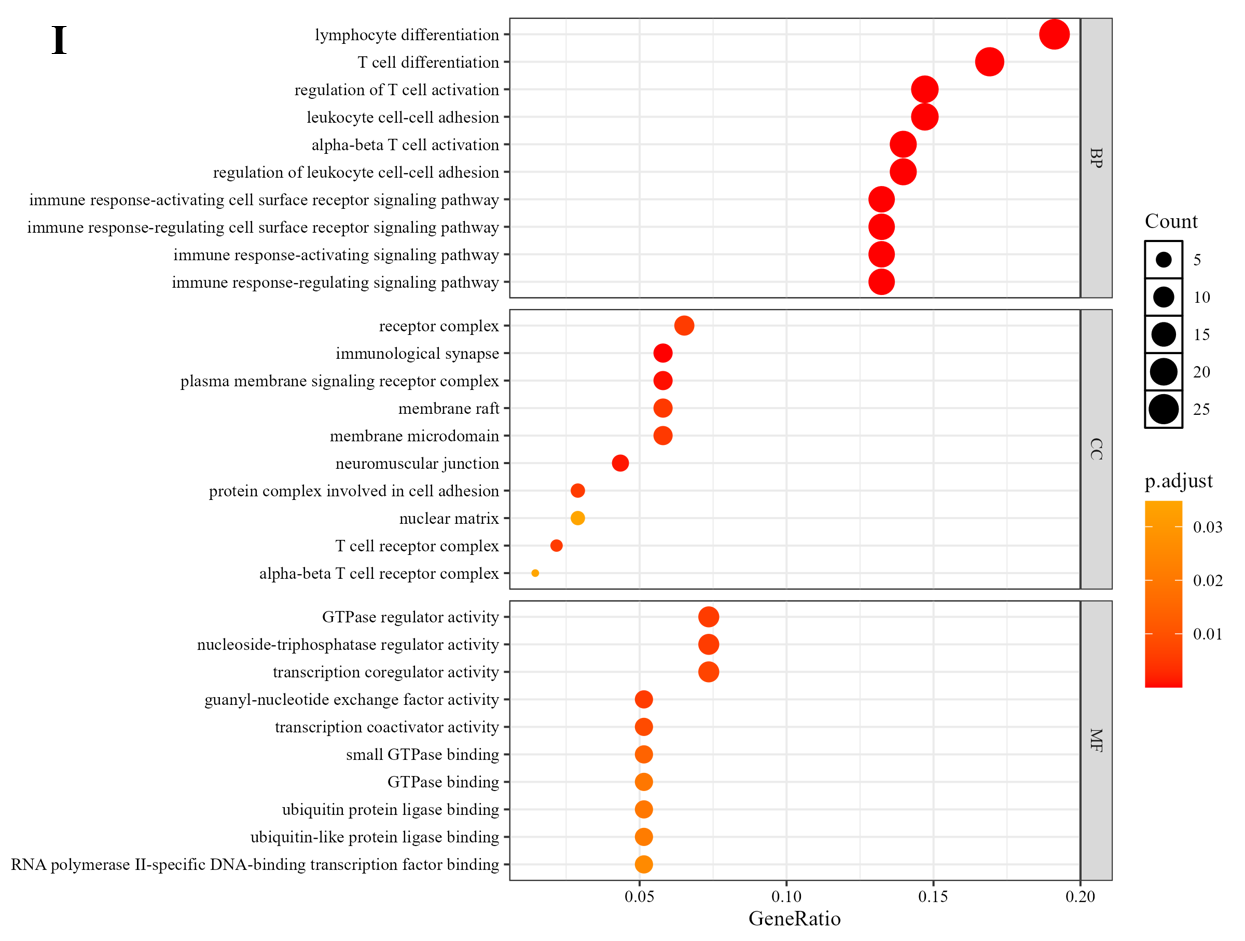


**
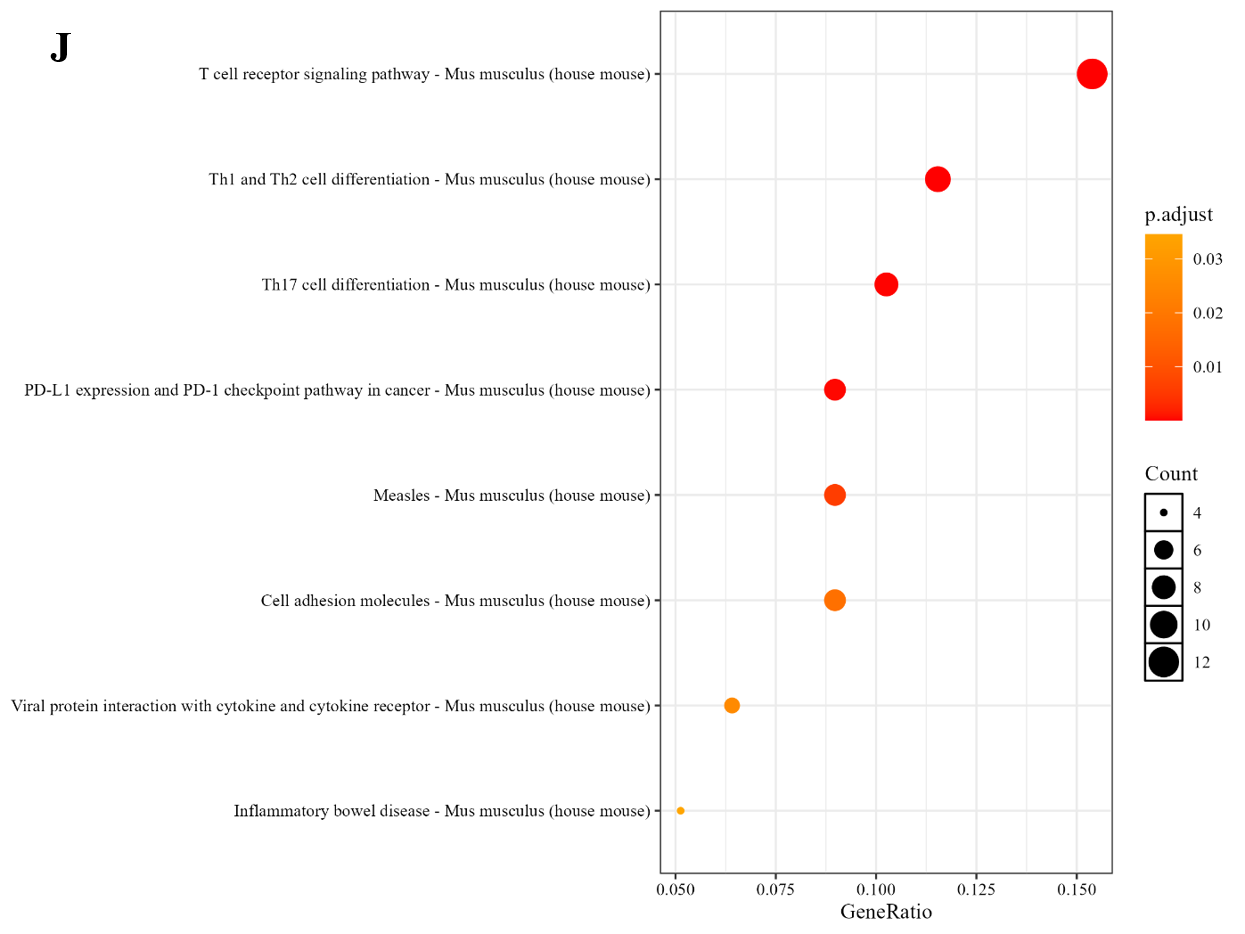
**

**Supplementary Figure 4. Single-cell transcriptomic data for the orthotopic glioma mouse model (GSE246154) from the GEO database and performed single-cell RNA sequencing analysis using R.** (A) UMAP visualization colored by cell type. (B) Heatmap showing relative expression of each of the 10 top expressed genes in each Leiden cluster. Columns correspond to cells, ordered by Leiden cluster. (C) UMAP visualization colored by T cell type from FigS4A. (D) Four different clusters and their specific marker gene expression levels, with brightness indicating log-normalized average expression, and circle size indicating the percent expressed.CD22 gene is marked in T cell type 04. (E) Dotplots shows marked T cell genes. (F) Violinplots shows T cell gene scores. (G) Dotplots shows IFN-γ related genes from T cell tpe. (H) Violinplots shows ifnar2 gene has higher scores in IFN-γ related genes. GO (I) and KEGG (J) analysis of T cells.

**
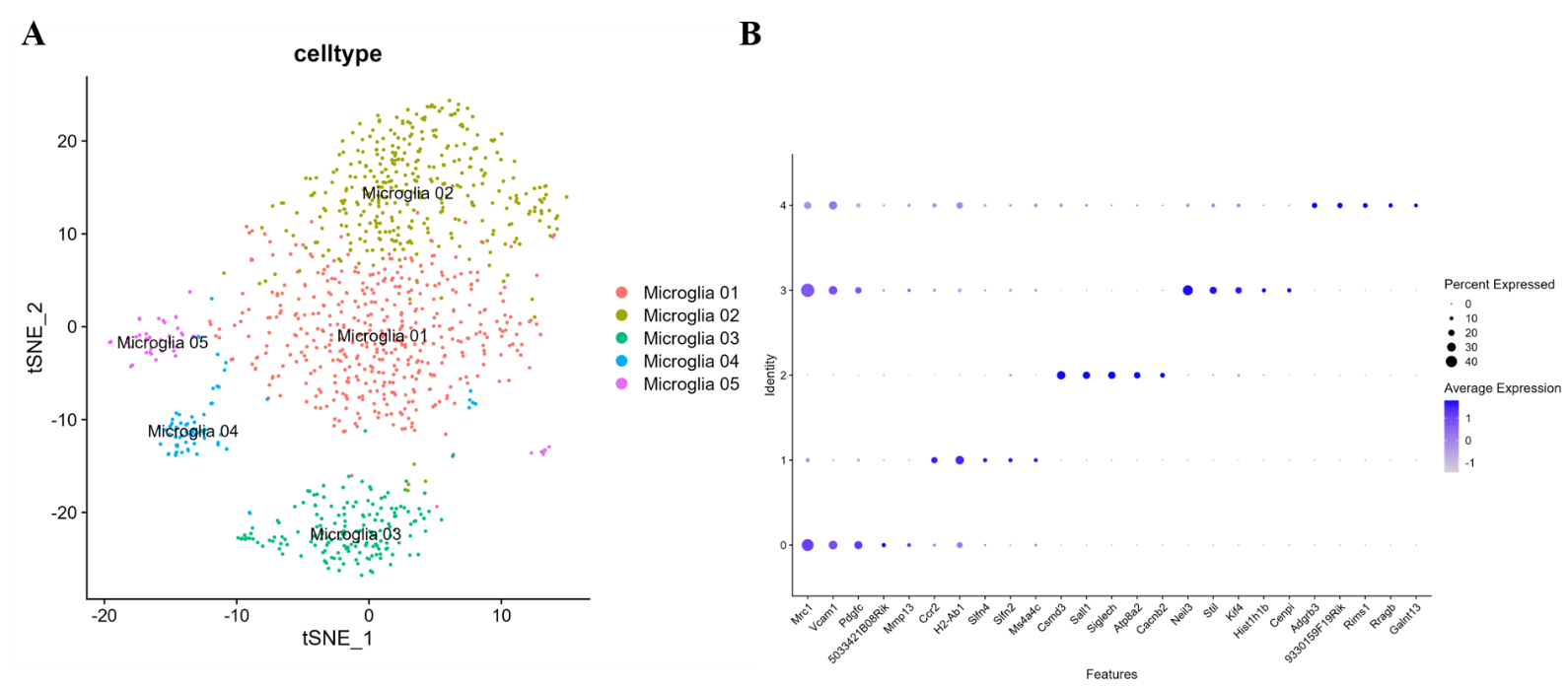
**


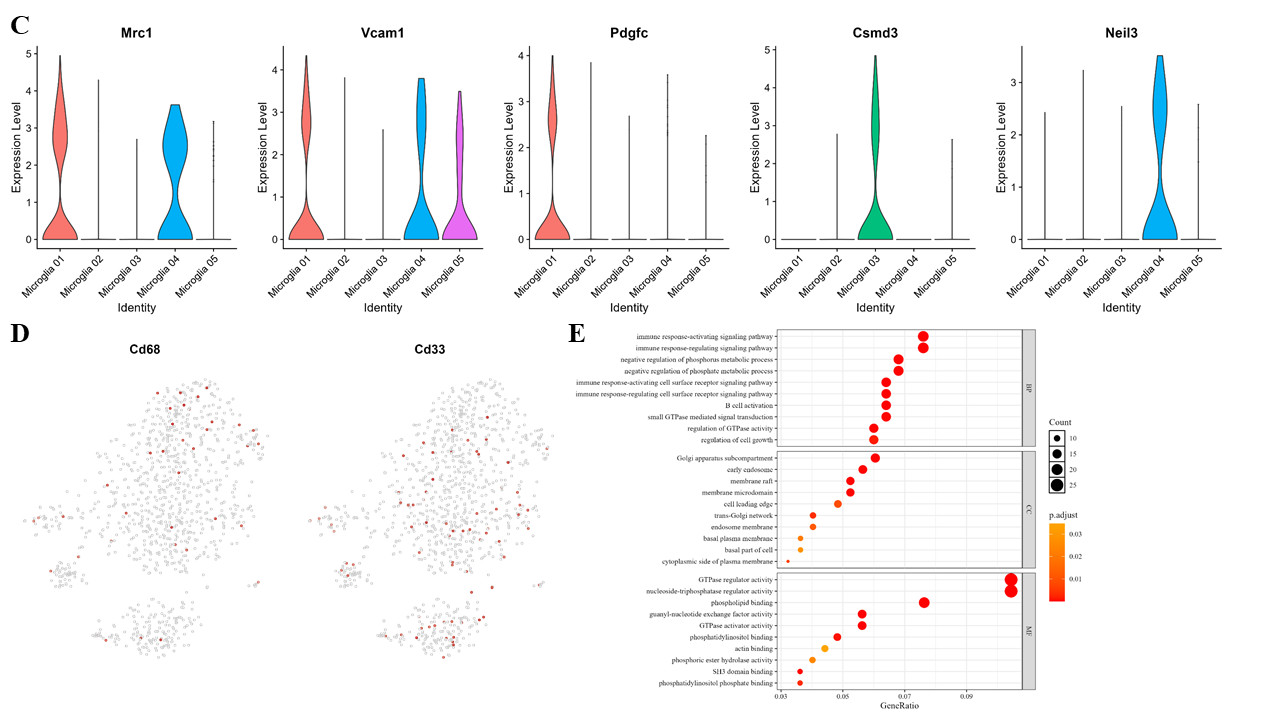

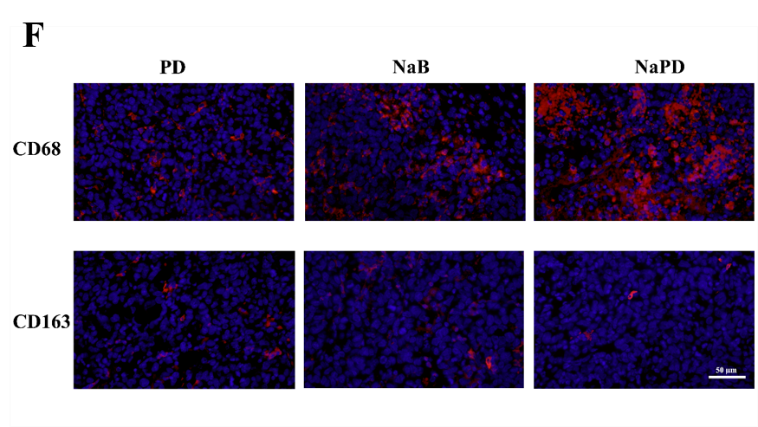


**Supplementary Figure 5. Single-cell transcriptomic data for the orthotopic glioma mouse model (GSE246154) from the GEO database and performed single-cell RNA sequencing analysis using R.** (A) UMAP visualization colored by Microglia cell type from Figure S5A. (B) Four different clusters and their specific marker gene expression levels, with brightness indicating log-normalized average expression, and circle size indicating the percent expressed. (C) Violinplots shows T cell gene scores. (D) Dotplots shows CD68 and CD33 cell genes. (E) KEGG analysis of Microglia cells. (F) Immunofluorescence staining with monoclonal antibodies against CD68 and CD163.
